# Cold stress alters transcription in meiotic anthers of cold tolerant chickpea (*Cicer arietinum* L.)

**DOI:** 10.1186/1756-0500-7-717

**Published:** 2014-10-11

**Authors:** Kamal Dev Sharma, Harsh Nayyar

**Affiliations:** Department of Agricultural Biotechnology, CSK HP Agricultural University, Palampur, 176062 HP India; Department of Botany, Panjab University, Chandigarh, India

**Keywords:** Anthers, *Cicer arietinum*, Cold stress, Cold tolerance, Gene expression, Male gametophyte, Pollen

## Abstract

**Background:**

Cold stress at reproductive phase in susceptible chickpea (*Cicer arietinum* L.) leads to pollen sterility induced flower abortion. The tolerant genotypes, on the other hand, produce viable pollen and set seed under cold stress. Genomic information on pollen development in cold-tolerant chickpea under cold stress is currently unavailable.

**Results:**

DDRT-PCR analysis was carried out to identify anther genes involved in cold tolerance in chickpea genotype ICC16349 (cold-tolerant). A total of 9205 EST bands were analyzed. Cold stress altered expression of 127 ESTs (90 up-regulated, 37 down-regulated) in anthers, more than two third (92) of which were novel with unknown protein identity and function. Remaining about one third (35) belonged to several functional categories such as pollen development, signal transduction, ion transport, transcription, carbohydrate metabolism, translation, energy and cell division. The categories with more number of transcripts were carbohydrate/triacylglycerol metabolism, signal transduction, pollen development and transport. All but two transcripts in these categories were up-regulated under cold stress. To identify time of regulation after stress and organ specificity, expression levels of 25 differentially regulated transcripts were also studied in anthers at six time points and in four organs (anthers, gynoecium, leaves and roots) at four time points.

**Conclusions:**

Limited number of genes were involved in regulating cold tolerance in chickpea anthers. Moreover, the cold tolerance was manifested by up-regulation of majority of the differentially expressed transcripts. The anthers appeared to employ dual cold tolerance mechanism based on their protection from cold by enhancing triacylglycerol and carbohydrate metabolism; and maintenance of normal pollen development by regulating pollen development genes. Functional characterization of about two third of the novel genes is needed to have precise understanding of the cold tolerance mechanisms in chickpea anthers.

**Electronic supplementary material:**

The online version of this article (doi:10.1186/1756-0500-7-717) contains supplementary material, which is available to authorized users.

## Background

Male gametophyte in flowering plants is a highly dynamic structure with active growth and high metabolic activity. It is an organ with highest sink strength in the flower and large amounts of sugars are transported to anthers to support its development and formation of pollen grains [1]. Anther is also the organ with high sensitivity to cold stress [2]. Within anther, the pollen development and pollen function under stress is the weakest link in plant sexual reproduction [3]. Pollen development proceeds through meiosis and sensitivity of the male gametophyte to stresses increases considerably after the onset of meiosis [4]. Pollen maturation is also one of the most sensitive stages [5]. Nutrition to young microspores and developing pollen grains is provided by the tapetum, which functions at maximum capacity to synthesize locular fluid [6]. At the same time, the pollen wall is also deposited on the developing pollen [6, 7]. Abiotic stress at the time of tapetum development aborts male gamete formation and results in sterile pollen [3, 8]. Cold stress perturb carbohydrate metabolism and alters anther morphology [8, 9]. As a whole, the temperature stress reduces pollen development, pollen fertility, anthesis, pollination and pollen tube growth [4, 10].

Chickpea (*Cicer arietinum* L.), a leguminous annual flowering herb, is grown for its protein rich grains in several parts of the world. The crop is a native of tropical Mediterranean region and is sensitive to chilling temperatures [11]. Temperatures below 15°C abort chickpea flowers and decrease the number of pods per plant and seeds per pod [9, 12–16]. Chilling stress prevailing during flowering and grain filling leads to nutritional deficiencies in the tapetum [13]. The susceptible genotypes show reduction in anther dehiscence, pollen load on the stigma, pollen germination and pollen tube growth [9, 17]. Growing tips of the pollen tubes also show distortions [9, 17] and fertilization is poor. Cold sensitivity in susceptible genotypes is manifested by increase in oxidative stress, increase in membrane damage, decrease in chlorophyll and relative leaf water content [15]. Flower abortion due to cold stress in chickpea is associated with lower levels of sucrose, glucose and fructose in anthers and pollen [13]. Of late, chickpea genotypes, ICC16348 and ICC16349, were found to be tolerant to cold [15]. These genotypes developed flowers and set pods at low temperatures. Cold tolerance in ICC16349 was manifested in the form of low electrolyte leakage and high chlorophyll and water content [15]. Total sugars and starch were found to be higher in cold tolerant genotypes compared to the susceptible ones whereas oxidative stress was low [15].

There is however, no study on identification or isolation of male or female gametophyte genes involved in reproduction or those involved in stress tolerance/susceptibility. Some transcriptomics studies on stress biology in chickpea organs other than anthers and gynoecium have been conducted [18–23]. The present study identified anther genes regulated differentially in response to cold stress in a cold-tolerant genotype. In addition, spatial and temporal expressions of selected genes in anthers, gynoecium, leaves and roots were also studied with the aim to identify organ specificity in gene expression under cold and to get an insight of gene regulation in different organs. To our knowledge, this is the first study on transcriptome of anthers in chickpea and other field legumes under cold stress conditions.

## Results

### Genome-wide expression analysis

**Figure 1:**
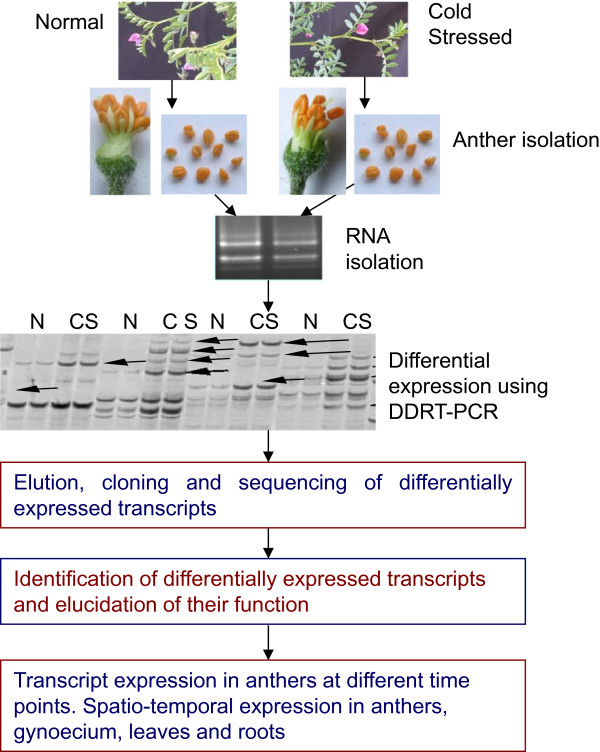
**Flow-chart showing the procedure to generate gene expression profiles.** DDRT-PCR was used to study differential transcript expression in cold-stressed chickpea anthers. The genotype used was ICC16349 (cold-tolerant) and the cold treatment was at 5 ± 1°C. Semi-quantitative RT-PCR was used to study temporal and spatial expression of selected genes. The cold stress treatment for DDRT-PCR included two time points (0 and 72 h). Temporal expression in anthers was studied at six time-points (0, 1.5, 12, 24, 72 and 120 h) and spatio-temporal expression at four time points (1.5, 12, 24, and 72 h). The organs used for spatio-temporal expression were anthers, gynoecium, leaves and roots.

To identify chickpea male gametophyte genes regulated differentially under cold stress, anther transcriptome of a cold-tolerant genotype (ICC16349) at 72 h post cold stress was compared with the transcriptome of anthers of plants growing under normal conditions (see Figure 1 for outline of the experimental procedure). Our previous studies have already established that ICC16349 possessed high degree of tolerance to cold and could flower and set seed under low temperature conditions [15]. DDRT-PCR generated a total of 10,567 bands. Bands smaller than 75 bases (1362 nos.) were rejected and remaining 9205 EST bands were analyzed. Of these 9205 EST bands, 206 were differentially regulated with more than two fold change in anthers of cold stressed plants. Among the differentially regulated bands, 133 were up-regulated (UP) and 73 down-regulated (DR). Sequence editing, clustering and contig analysis revealed that expression of only 127 ESTs [90 (70.9%) UP; 37 (29.1%) DR was altered due to cold stress. The proportion of UP genes compared to DR ones was 2.4 indicating that cold-tolerance in anthers of ICC16349 was manifested primarily by the enhanced expression of genes.

### Functional analysis of differentially expressed genes

**Figure 2:**
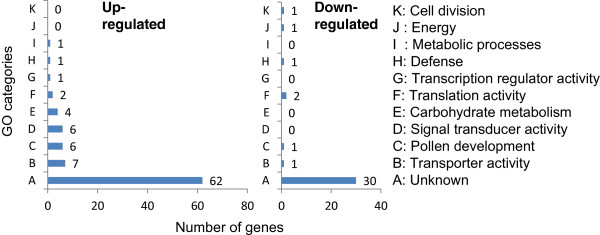
**Functional categorization of cold responsive anther genes in ICC16349.** The number of genes were finalized using the filtering criteria of fold change >2.0 and P-value correction ≤0.05 by FDR (Benjamini-Hochberg). Genes were classified into 11 different functional categories based on gene ontology. Number of genes in each category are presented at the termini of bars.

Information on identity, cellular component, biological and molecular functions of all the genes was obtained form NCBI-BLAST, Gene Ontology (GO) analysis and KEGG pathway (Additional file 1). Based on GO analysis, the genes were classified into three main GO categories, the biological process (BP), cellular component (CC) and molecular function (MF). Of the 127 genes, only 35 (27.5%, 28 UP, 7 DR) could be functionally characterized according to GO descriptions (Table 1). The remaining 92 ESTs (more than two third) were of unknown MF and BP (Additional file 1). The ESTs with altered expression were further assigned into 10 GO subcategories (Figure 2, Table 2). Among these 10 subcategories, those related to ion transport, pollen development, signal transduction and carbohydrate metabolism appeared to be more important for cold tolerance because the number of altered transcripts in these subcategories were more. Twenty five of the functionally characterized transcripts (71.4% of transcripts with GO description) belonged to these four subcategories. Besides more number of genes, other unique feature was up-regulation of majority of the transcripts (23 out of 25, 92%) in these subcategories. Only two genes in these subcategories were repressed, one each for ion transport and pollen development. The GO subcategories with relatively less number of genes were translation, transcription, energy, cell division and metabolic processes. The genes in these subcategories did not show a definite trend as some of those were UP whereas others were DR.

**Table 1:** **Gene ontology score-based categorization of differentially regulated transcripts in anthers of ICC16349**

	Up-regulated	Down-regulated	Total
Total	90	37	127
BP	25	6	31
CC	16	5	21
MF	26	5	31

BP, biological process; CC, cellular component; MF, molecular function.

**Table 2:** **Functional characterization of 35 differentially expressed transcripts from the DDRT-PCR**

Transcript no	Homology and e-value	Biological function	Molecular function	Cellular component
**Transporter activity genes**				
A3*	Cation/H^+^ antiporter 14, *Arabidopsis thaliana,* AT1G06970, 4.1e-05	Ion transport	Antiporter activity	Membrane
A20*	Cation efflux system protein, *Agrobacterium radiobactor* K84, YP_002543585.1, 0.33	Ion transport, transmembrane transport	Copper ion binding	Membrane
A82-1*	Heavy metal efflux pump CzcA, *Gamma proteobacterium*, ZP_05061697.1, 4e-03	Unknown	Cation transmembrane transporter activity	Unknown
A116-3*	L-ascorbate oxidase like protein, *M. truncatula*, XP_003611827.1, 4e-05	Ion transport	Copper ion binding	Unknown
A121-1*	AT5G57110 (Ca2+ transporting ATPase), *A. thaliana*, BAH20100.1, 6e-05	Calcium transport, ATP biosynthetic process	Calcium ion transport	Membrane
A123-1*	Potassium channel tetramerization domain-containing protein, *R. communis*, XP_002509821.1, 3e-29	Ion transport	Voltage-gated potassium channel activity	Membrane
A125-1*	F16A14.19, *A. thaliana*, AAF79412.1, 8e-20	Transport	Anion channel activity	Unknown
A118**	ABC transporter family, *M. truncatula*, XP_003590459.1, 8e-27	Unknown	Ttransporter activity	Plasmodesmata
**Translation**				
A22*	40S ribosomal protein SA, *M. truncatula*, XP_003638087.1, 4e-3	Translation	Ribonucleoprotein	Cytoplasm
A73**	60S ribosomal protein L27a-3, *M. Truncatula,* XP_003613127.1, 2e-16	Translation	Structural constituent of ribosome	Ribosome
AC39GA2*	Translation initiation factor EIF-2B epsilon, *M. truncatula,* XP_003618849.1, 3e-06	Translation	Translation initiation factor activity	Unknown
AC41GF1**	60S ribosomal protein L34, *M. truncatula,* XP_003621181.1, 6e-09,	Translation	Ribnucleoprotein	Large subunit of ribosome
**Transcription regulator**				
AC 47G E1*	SRCI, *Glycine max*, BAA19768.1, 0.096	Cold stress regulation	Transcription	Unknown
**Carbohydrate metabolism**				
A36-2*	Beta-galactosidase, *Arabidopsis thaliana*, CAB64750.1, 4e-3	Carbohydrate metabolism, pollen development	Beta-galactosidase activity	Apoplast
A59-2*	Glycerol kinase, *Glycine max*, NP_001237303.1, 1e-21	Glycerolipid metabolism	Glycerol kinase activity	Unknown
A102-2*	Aconitate hydratase, *M. truncatula*, XP_003612247.1, 4e-24	Carbohydrate metabolism (converts citrate to isocitrate)	Iron sulfur cluster binding	Cytoplasm
AC44GA 2*	Sucrose phosphorylase, *Vibrio harveyi* HY01, ZP 01985256.1, 0.64	Starch and sucrose metabolism	Cation binding, sucrose phosphorylase activity	Unknown
**Pollen development**				
A10*	Peroxisomal ABC transporter, *M. truncatula*, XP_003601968.1, 1e-10	Transport (fatty acids), Pollen tube elongation, ovule fertilization, and seeds germination after imbibition	ATP binding	Glyoxisomal membrane
A60*	Pectin methylesterase, *M. truncatula*, XP_003595372.1, 7e-17,	Cell wall modification, tetrad separation, pollen tube growth	Pectin methylesterase activity	Membrane
A99-1*	Microspore-specific promoter2, *Arabidopsis thaliana*, NP_5686669.1, 0.02	Pollen development	Transcription	Chloroplast
A101*	Pectin esterase, *M. truncatula*, XP_003591164.1, 1e-06	Cell wall modification, pollen tube growth	Pectin methylestera activity	Cell wall
A104-2*	SYP124 (SYNTAXIN OF PLANTS); SNAP receptor, *M. truncatula*, XP_003593444.1, 1e-3	Vesicular mediate transport, intracellular protein transport, pollen development	SNAP receptor activity	Membrane
AC52GD1*	Protein WAX2, *M. truncatula*, XP_003606194.1, 5e-28	Pollen sperm cell differentiation	Iron ion binding, fatty acid biosynthetic process	Integral to membrane
A64-1**	Early nodulin-like protein, *M. truncatula*, XP_003609073.1, 8e-04	Pollen development	Copper ion binding	Membrane
**Signal transducer activity**				
A67*	Cysteine-rich receptor-like protein kinase, *M. truncatula,* XP_003589476.1, 2e-3	Calcium-mediated Signal transduction, pollen development, recognition of pollen	Protein serine/threonine kinase activity	Membrane
A81*	Protein kinase serine/threonine, *A. thaliana*, CAA16700.1, 1e-37	Signal transduction	Protein serine/threonine kinase activity	Nucleus
A97-2*	Ralf-like 19 protein, , *A. thaliana*, NP_850219.1, 5e-25	Signal transduction	Unknown	Unknown
A120-2*	Serine/threonine protein kinase, *M. truncatula*, XP_003618563.1, 2e-3	Signal transduction	Protein serine/threonine kinase Activity	Unknown
A140-2* (pollen development)	Cyclin-dependent kinase CDC2C, *M. truncatula*, XP_003621316.1, 2e-36	Signal transduction, pollen tube growth	Serine/threonine protein kinase	Unknown
AN59CA2*	Casein kinase, *Ricinus communis*, XP_002516524.1, 5e-17	Signaling transduction	ATP binding	Unknown
**Defense**				
A126-1*	Wound responsive protein, *Phaseolus vulgaris*, Q09020.1, 7e-07	Defense	Unknown	Unknown
A114**	RRP1, *Medicago truncatula*, AB1511616.1, 0.1e-4	Defense, resistance to *Peronospora parasitica*	Unknown	Unknown
**Energy**				
AC45GA3**	ATPase subunit 8, *Lotus japonicus*, YP_005090498.1, 2e-71	Energy	Hydrogen ion transmembrane transporter activity	Mitochondria
**Metabolic processes**				
A98-2*	Hydrolase, *Zea mays*, NP_001150070.1, 1e-6	Unknown	Hydrolase activity	Unknown
**Cell division**				
A71-1**	Cell division cycle and apoptosis regulator protein, *M. truncatula*, XP_003613873.1, 5e-07	Cell division	Unknown	Unknown

Homologies are as per BLASTX.

*Up-regulated, **Down-regulated.

The subcategory pollen development had maximum number (ten) of altered transcripts. Seven of these are listed under subcategory pollen, one (beta-galactosidase) under carbohydrate metabolism and two (cysteine-rich receptor-like protein kinase and CDC2C) under signal transduction (Table 2). As per GO and available literature, the BP of these genes were tetrad separation and pollen release [pectin methylesterase (PME), pectin esterase (PE)], pollen development [SNAP receptor, protein WAX2, early nodulin-like protein (ENODL6), beta-galactosidase] and pollen tube growth (peroxisomal ABC transporter, PME and PE). The MF of pollen specific transcripts were signal transduction, transcription, cell wall modification, protein transport, fatty acid transport and ion binding (Table 2). Except one gene (ENODL6), all genes in this subcategory were UP. Carbohydrates are considered vital for normal pollen development and four carbohydrate metabolism genes i.e. beta-galactosidase, glycerol kinase, aconitate hydratase and sucrose phosphorylase were up-regulated (Table 2). The function of these genes is to release free sugars from complex carbohydrates or triacyglycerol (glycerol kinase).

**Figure 3:**
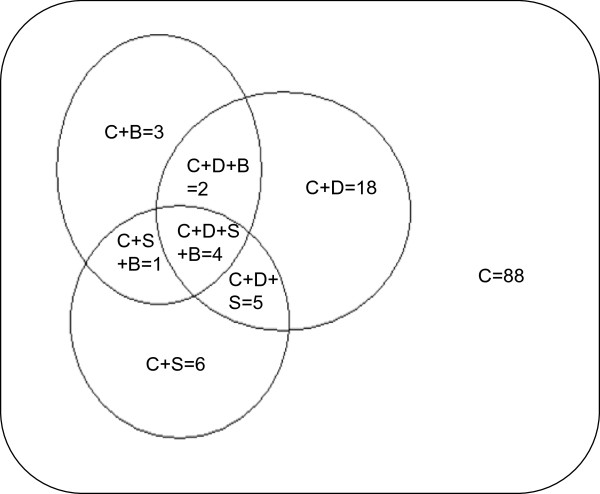
**Number of cold-responsive transcripts which are common to other stresses.** The stresses were C, cold; D, drought; S, salinity; B, biotic stress. The drought, salinity and biotic stress responsive chickpea ESTs in databases that had homology to cold tolerance responsive transcripts (present study) were identified using NCBI-BLASTN. The number of transcripts which were common to at least one stress other than cold was 39.

Additional information on differentially-regulated genes was also obtained from NCBI-BLAST (Additional file 1). Of the 127 differentially regulated chickpea ESTs identified in the present study, only 40 had similarity to chickpea ESTs listed in the databases (EST database, http://www.ncbi.nlm.nih.gov/nucest?term=chickpea). The remaining 87 ESTs were new records for chickpea. Large proportion of new ESTs identified in the present study might be attributed to the source organ used i.e. anther. This is the first study on chickpea anther transcriptome. In earlier studies, the leaf, stem and bud transcriptomes were analyzed [18–23]. The analysis revealed that 18 of the 40 ESTs were common between cold and drought, 6 between cold and salinity, 3 between cold and biotic stresses, and 5 among cold, drought and salinity (Figure 3). Four of the ESTs were common to all the four stresses i.e. cold, drought, salinity and biotic.

### Transcript expression at different time points in chickpea anthers

**Figure 4:**
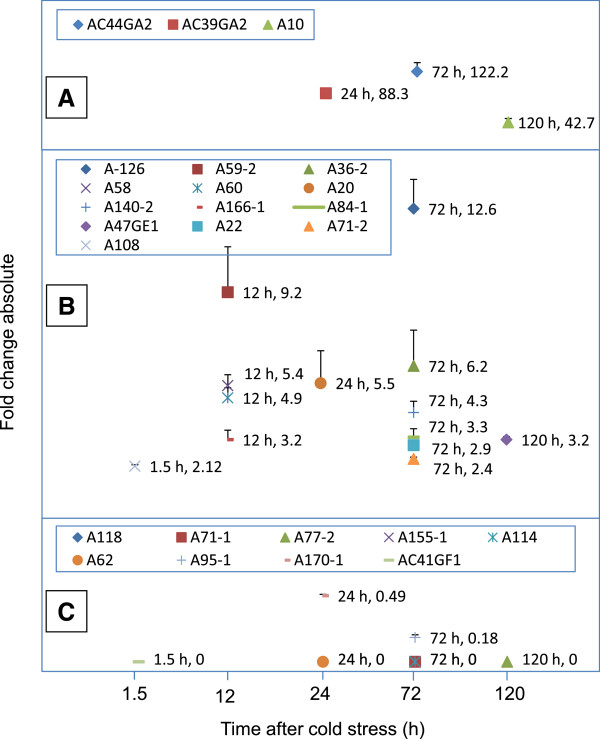
**Time after onset of cold stress at which the up-regulated genes showed maximum expression and down-regulated genes the maximum repression.** The expression in up-regulated genes decreased after this time point whereas in down-regulated genes it either remained the same or increased. The expression in anthers of ICC16349 was studied using RT-qPCR at six time points (0 h, 1.5 h, 12 h, 24 h, 72 h and 120 h) after cold stress and is presented as fold change absolute compared to expression at 0 h. Chickpea actin gene acted as control and was used for normalization of expression data. The functions of the ESTs as per gene ontology are A140-2: signal transduction; AC47GE1: transcription; A36-2, A59-2, AC44GA2: carbohydrate metabolism; A10, A60: pollen development; A20, A118: transport; A114, A126-1: defense; A22, AC39GA2, AC41GF1: translation; A71-1: Cell division; A58, A62, A71-2, A77-2, A84-1, A95-1, A108-2, A155-1, A166, A170-1: unknown function. Panel **(A)**, **(B)** show up-regulated and panel **(C)** down-regulated genes, respectively.

To confirm the DDRT-PCR expression profile and to study gene regulation at the beginning of cold stress and at various time points thereafter, reverse transcription quantitative polymerase chain reaction (RT-qPCR) analysis of anthers of the cold-tolerant genotype was carried out at six time points (0 h, 1.5 h, 12 h, 24 h, 72 h and 120 h) after cold stress. A representative set of 25 ESTs belonging to 9 GO categories (signal transduction, transcription, carbohydrate metabolism, pollen development, transport, defense, translation, cell division and unknown function) and having both the UP (16 nos.) and DR (9 nos.) transcripts was selected for RT-qPCR analysis (Additional file 2). These ESTs, in RT-qPCR, had differential expression similar to DDRT-PCR. In the category of UP genes, three transcripts i.e. AC44GA2 (sucrose phosphorylase, 122.2 fold at 72 h), AC39GA2 (translation initiation factor EIF-2B epsilon, 88.3 fold at 24 h) and A10 (peroxisomal ABC transporter, 42.7 fold at 120 h) showed maximum increase over the untreated control (Figure 4A, Additional file 3). Other highly UP transcripts were A-126 (wound-responsive gene; 12.6 fold at 72 h), A59-2 (glycerol kinase; 9.2 fold at 12 h), A36-2 (beta-galactosidase; 6.2 fold at 72 h), A58 (unknown; 5.4 fold at 12 h) and A60 (PME; 4.9 fold at 12 h, Figure 4B). Among DR transcripts, the expression of seven transcripts out of nine declined to zero at different time points after cold stress (Figure 4C). Only one transcript i.e. AC41GF1 (translation) showed quick decline in expression to zero following 1.5 h of cold stress.

**Figure 5:**
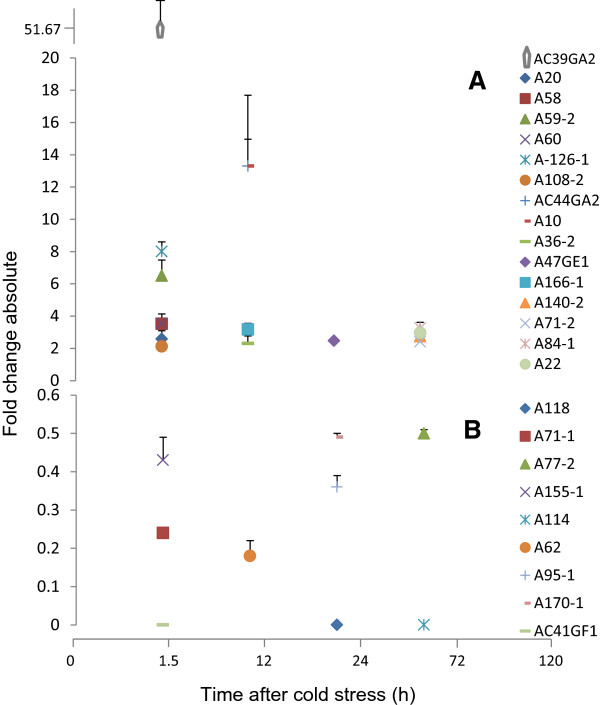
**Time after onset of cold stress at which the transcripts showed >2 fold change in expression.** The expression using RT-qPCR was studied at six time points (0 h, 1.5 h, 12 h, 24 h, 72 h and 120 h) after initiation of cold stress and is presented as fold change absolute compared to expression at 0 h. Chickpea actin gene acted as control and was used for normalization of expression data. The functions of the ESTs as per gene ontology are A140-2: signal transduction; AC47GE1: transcription; A36-2, A59-2, AC44GA2: carbohydrate metabolism; A10, A60: pollen development; A20, A118: transport; A114, A126-1: defense; A22, AC39GA2, AC41GF1: translation; A71-1: Cell division; A58, A62, A71-2, A77-2, A84-1, A95-1, A108-2, A155-1, A166, A170-1: unknown function. Panel **(A)** shows up-regulated and panel **(B)** down-regulated genes, respectively.

Time point expression also allowed us to identify the time at which the genes showed more than two- fold increase/decrease (Figure 5). The UP genes with more than two fold increase within 1.5 h were termed as ‘early-on’ whereas those with such increase at 72 h or 120 h were termed as ‘late-on’. The genes with more than two fold increase at 12 h or 24 h were called ‘intermediate’. Eight genes were ‘early-on’ , four ‘intermediate’ and four ‘late-on’ (Figure 5A, Additional file 3A, C). The role of some of the transcripts (A20, A108-2, A166-1, A36-2 and A59-2) appeared to be restricted only to early stages of cold tolerance (Additional file 3A) as their expression declined to the levels of non-stressed anthers by 72 or 120 h. Among the DR genes, A71-1 (cell division), A155-1 (energy) and AC41GF1 (translation) were early in down-regulation; A62 (unknown), A95-1 (unknown), A170-1 (unknown) and A118 (transport) were ‘intermediate’; and A114 (defense) and A77-2 (unknown) were late in down regulation (Figure 5B, Additional file 3B). Thus, the regulation of expression whether that of UP or DR genes was in a time-limited manner.

### Spatial and temporal expression of genes in anthers, gynoecium, leaves and roots

The expression of the 25 genes, which were used for time point expression studies in anthers, was also elucidated in anthers, gynoecium, leaves and roots (Additional file 4, Figure 6). The aim was to understand gene regulation under cold stress in different chickpea organs and to identify organ specificity in gene expression. The differential gene regulation in different organs at different time points was evident. The expression of five transcripts, A166 (unknown), glycerol kinase (A59-2), PME (A60), translation (A22), and A108-2 (unknown) was significantly higher in anthers compared to other organs indicating their anther-specificity and their important role in anther development under cold. The transcript A166 expressed only in anthers but not in other organs except at 72 h when it expressed in roots too. Three transcripts i.e. cation efflux protein (A20), A71-2 (unknown) and A58 (unknown) had very high expression in leaves compared to other organs (Additional file 4) whereas peroxisomal ABC transporter (A10) it did not express in roots at any time point. The transcript A126 was root-specific as it had higher expression in roots compared to other organs.

**Figure 6:**
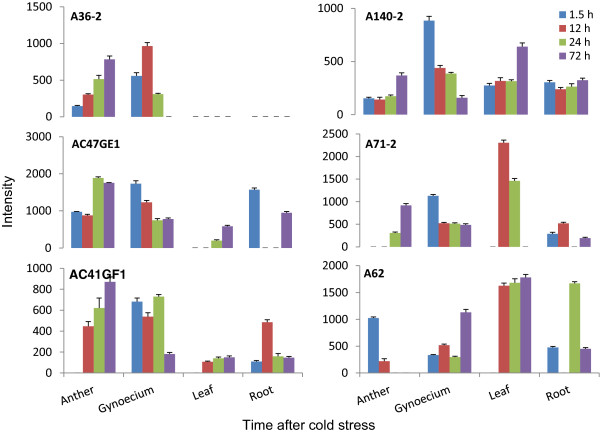
**Differential gene expression in cold-stressed anthers and gynoecium of ICC16349.** Only those genes with contrasting expression patterns in terms of increase or decrease over time between the two organs are presented. The comparative expression in leaves and roots is also presented. All the four organs were harvested from same set of plants. The expression using RT-qPCR was studied at four time points (1.5 h, 12 h, 24 h and 72 h) after cold stress and is presented as intensity of the RT-qPCR bands. Chickpea actin gene acted as control and was used for normalization. The functions of the ESTs as per gene ontology are A140-2: signal transduction; AC47GE1: transcription; A36-2: carbohydrate metabolism; AC41GGF1: translation; A62, A71-2: unknown function.

All anther genes except one i.e. A166 expressed in gynoecium. This pointed towards high degree of commonality in genes involved in cold tolerance in the two reproductive organs i.e. anther and gynoecium. Two of the genes (A36-2, A114) expressed only in anther and gynoecium and not in other organs. The commonality of cold-responsive genes in anthers and gynoecium did not mean similar patterns of gene regulation. Only 9 out of 25 genes have similar patterns of gene regulation between the two organs (Additional file 4). The remaining 16 genes have contrasting patterns of regulation. While the expression of these genes in one of the organs was high, in other, it was low. Among these 16 genes, a group of 6 genes had unique patterns of regulation between the two organs (Figure 6). These genes, in one of the organs, showed increase in expression over time, while in other there was gradual decrease over time. For example, the expression of A36-2 in gynoecium was 3.8 times to that in anthers at 1.5 h, it decreased to 0.6 times at 24 h and the gene was switched off in gynoecium by 72 h. In contrast to this, the expression in anthers was low at 1.5 h but it increased steadily over time and reached a peak at 72 h, the time by which it was switched off in gynoecium. The other genes in this group were A140-2, AC47GE1, AC41GF1, A62 and A71-2. All these genes except A62 showed up-regulation over time in anthers and down-regulation in gynoecium (Figure 6).

## Discussion

### DDRT-PCR identified novel genes for cold tolerance in anthers

Male gametophyte is the most sensitive chickpea organ to cold stress. Microsporogenesis and subsequent pollen development are affected adversely when chickpea plants are exposed to temperatures below 10°C [2]. Cold-hardy chickpea genotypes on the other hand maintain normal anther and pollen development leading to pod formation and seed set [15]. The present study revealed 127 differentially expressed transcripts in tolerant genotype of chickpea under cold stress including 92 (72.4%) novel ones for which GO descriptions are not available. It appears that induction of cold tolerance in chickpea is regulated by a relatively small number of genes (present study, [22]). Similar to our study, the number of transcripts (1%; 96 out of 7300) with altered expression was also less in meiotic anthers of heat stressed but heat-tolerant tomatoes [24]. The comparison between heat tolerant and heat susceptible tomato genotypes vis-à-vis number of differentially expressed genes under heat stress was also made [24]. The number of altered transcripts was almost same in both types of the genotypes but the patterns of gene regulation were different. While majority of the genes were UP in the tolerant genotype of tomato, majority were DR in the susceptible one [24]. Similar pattern of gene regulation was also observed in chickpea anthers (present study) where more than two third (70.9%) of the altered transcripts were UP as a result of cold stress in the tolerant chickpea genotype. Gene regulation under cold stress in tolerant Arabidopsis and susceptible sunflower was also similar to that observed in tomato and chickpea [25, 26].

### Temporal and spatial gene regulation during cold stress tolerance

Spatial and temporal control of gene expression is crucial for the development of different plant organs including anthers [27]. The regulation of chickpea anther genes under cold stress was also in a time limited manner. For example, the expression pattern of genes for cell wall, carbohydrate metabolism and fatty acid metabolism matched to the physiological development of pollen during early stages. At the time of onset of cold treatment, the chickpea anthers had microspores in the tetrad stage [present study]. Rapid and highly orchestrated developments leading to mature pollen development take place in anthers after tetrad formation [28]. Some important morphological/physiological features during this phase are separation and expansion of microspores, pollen mitosis I, pollen mitosis II and pollen maturation. During these stages, the tapetum cells feed nutrients to developing microspores [29]. The genes with BP as tetrad separation (PME), pollen expansion by cell wall loosening (beta-galactosidase) and triacylglycerol metabolism leading to sucrose synthesis (glycerol kinase) were up-regulated within 1.5 h of cold stress. Early expression of these genes matched with the morphological features of pollen development i.e. tetrad separation and microspore expansion. Since, tapetum stores lipids which are utilized by rapidly developing microspores in anthers [30, 31], glycerol kinase might be the possible enzyme to convert tapetum lipids to sucrose. The genes, sucrose phosphorylase (BP: production of free sugars from sucrose, KEGG Pathway) and peroxisomal ABC transporter (BP: pollen maturation, pollen exine formation and male fertility, MF: transportation of fatty acids for ß-oxidation, [32, 33]) over-expressed later than early UP genes. It might be possible that sucrose produced by the glycerol kinase is the target molecule for sucrose phosphorylase and peroxisomal ABC transporter supplies necessary triacylglycerols for action by glycerol kinase. The up-regulation of peroxisomal ABC transporter up to 120 h indicated its possible role till later stages of pollen development.

### Carbohydrate metabolism: an important part of cold tolerance mechanism in chickpea anthers

The DDRT-PCR, time point and spatial expression data pointed towards the prominent role of carbohydrate metabolism in cold tolerance by anthers of the tolerant chickpea genotype ICC16349. In the present study, all four genes related to carbohydrate metabolism showed over-expression in cold-stressed anthers. Among these genes, the sucrose phosphorylase catabolizes sucrose to yield fructose and glucose (KEGG pathway), the beta-galactosidase acts on beta-galactosides and the aconitate dehydratase catalyzes conversion of citrate to isocitrate in the tricarboxylic cycle through which energy is generated and precursors for important biomolecules are synthesized (KEGG pathway). The glycerol kinase converts triacylglycerols to sucrose, a substrate for sucrose phosphorylase. A transporter of triacylglycerols (peroxisomal ABC transporter) was also UP. Outcome of overexpression of these genes would be the production of higher amounts of free sugars (sucrose, glucose and fructose) that in plants provide necessary energy and carbon skeleton for growth. Rapid microspore/pollen developments after meiosis also need higher amounts of energy and carbon [1–3]. It appears that the tolerant ICC16349 ensures adequate free sugar accumulation by enhancing the expression of carbohydrate and fatty acid metabolism genes. The free sugars might also serve another purpose as osmolytes and cryoprotectants. Physiological studies in cold-susceptible plants of chickpea have revealed that the disruption of sugar metabolism is the cause of cold induced pollen sterility [3, 13]. Decreased carbohydrate supply is one of the major factors for cold-induced pollen sterility in susceptible genotypes of several crops including chickpea [3, 13, 15, 34]. Cold-treated susceptible plants accumulate low levels of free sugars and flowers of such plants abort [13]. On the other hand, soluble sugars enhance cold stress tolerance in cold-hardy plants [34]. Comparison between cold-susceptible and tolerant plants showed that the leaves of cold-treated tolerant chickpea genotypes had higher amounts of sugars than the treated susceptible ones [15].

### Pollen development genes: additional mechanism of viable pollen formation under cold stress?

Accumulation of free sugars in anthers was considered to be the major mechanism for formation of viable anthers/pollen in chickpea as well as other crops [3, 13, 15]. Our study, however, pointed towards the possibility of occurrence of additional mechanisms involving pollen-development specific genes. Of the genes with known function, 28.6% are involved in pollen development and pollen tube growth in other crops (Table 2). It is well established that separation of tetrad during microsporogenesis requires loosening of cell wall. At least three genes with possible role in cell wall loosening and release of pollen grains from tetrad were UP in the present study. These were PME, PE and beta-galactosidase. The PME has been shown to loosen cell walls leading to pollen release [35, 36] and in Arabidopsis mutated for this gene, pollen grains were released as tetrad [35]. Similarly, beta-galactosidase was associated with pollen expansion after microspore meiosis [37, 38]. Microsporogenesis followed by pollen development is a metabolically very active phase in plant reproduction [2, 3] and requires continuous supply of wall and other materials. It might be possible that the genes for intracellular protein transport (SYP124, vesicular mediated transport) and fatty acid transport (peroxisomal ABC transporter) UP in the cold-treated anthers, fulfill this requirement. It is already established that the peroxisomal ABC transporter plays role in pollen maturation, pollen exine formation and pollen tube growth [32, 33] whereas SYP124 in pollen tube growth [39]. Another gene, *WAX2* is required for fertility and seed formation in Arabidopsis [40]. The *wax2* mutants suffered from severe pollen sterility and seedlessness, at least under low humidity conditions [40]. ENODL6 is also involved in pollen development [41]. In addition to this, two signal transducers (cysteine rich receptor-like protein kinase and CDC2C) with BP as pollen development (GO description) were also UP. It appears that anther development in cold-hardy ICC16349 under cold stress is due to accumulation of free sugars and osmolytes (present study, [15]). On the other hand, the viable pollen development under cold stress might involve both the pollen development and carbohydrate metabolism genes. Further studies are, however, needed to support this hypothesis.

## Conclusions

In this study, a global view of gene expression in anthers of a cold-tolerant genotype during cold stress was obtained. This is the first study on transcriptome of chickpea anthers and it revealed that relatively less number of anther transcripts were altered in cold- tolerant chickpea as a result of cold stress. More than two third of the differentially regulated transcripts were novel with unknown BP and MF. Another unique feature was up-regulation of majority of the altered transcripts. Pollen development, transport, signal transduction and carbohydrate metabolism were the four important GO subcategories comprising 25 of the 35 functionally characterized transcripts. The expression of altered genes over time in cold stressed anthers revealed that some genes over-expressed immediately after onset of stress while others took several hours or days to do so. Spatio-temporal transcript expression involving four organs and four time points revealed differences in gene regulation in anthers, gynoecium, leaves and roots as a result of cold stress. The patterns of gene regulation in anthers and gynoecium were interesting. Though, all genes except one were common in these two organs, the expression patterns of majority of the genes were contrasting. While the expression in one organ increased with time after cold stress, the expression in other organ decreased. The study pointed towards the existence of dual cold tolerance mechanism operating in tolerant chickpea anthers. While the anthers were protected by enhancing triacylglycerol and carbohydrate metabolism, normal pollen development appeared to be ensured by regulating pollen development and carbohydrate metabolism genes. Chickpea is also affected by abiotic stresses other than cold [42]. In chickpea, there exists a cross talk among genic responses to abiotic (drought, cold, high salinity) and biotic (fungal pathogen *Ascochyta rabiei*) stresses [43, 44]. To breed multi-stress tolerant chickpea, there is a need to identify shared as well as unique genes/responses leading to viable pollen development under different abiotic stresses.

## Methods

### Plant material, growth conditions and stress treatment

Chickpea genotype ICC16349 (cold-tolerant) was grown in the greenhouse at 25 ± 1°C/22 ± 1°C (12 h day/12 h night cycles) with approximately 50-70% relative humidity until flowering. The plants were illuminated (16 h/8 h light/dark cycle) using overhead white fluorescent tubes (300 μmol m^-2^ s^-1^). In each 10" diameter plastic pot filled with soil, sand and vermicompost (1:1:1), two plants were grown. At a fixed time in the day, the flowers at three days pre-pollen dehiscence stage were tagged and the plants were shifted to a cold chamber (Blue Star, 5 ± 1°C, humidity 50-60%) illuminated with overhead white fluorescent tubes (16 h light/8 h dark cycle). To identify cold tolerance genes, three separate experiments were conducted, i) differential display reverse transcriptase polymerase chain reaction (DDRT-PCR) to identify differentially regulated genes in ICC16349 under cold stress, ii) gene expression in anthers of ICC16349 at different time points using RT-qPCR and iii) temporal and spatial expression of genes in anthers, gynoecium, leaves and roots using RT-qPCR (see Figure 1 for flowchart of experimental procedure). The duration of cold stress for DDRT-PCR experiment was 0 h and 72 h. For time point expression of genes in anthers, cold stress was provided for 0 h, 1.5 h, 12 h, 24 h, 72 h, 96 h and 120 whereas for the spatio-temporal gene expression studies, the duration of stress was 1.5 h, 12 h, 24 h and 72 h. The organs from cold-treated plants were harvested within the cold chambers and stored immediately in liquid nitrogen (-196°C) until RNA isolation. The plants growing at 25 ± 1°C/22 ± 1°C and not subjected to cold stress acted as untreated control.

### RNA isolation and first strand cDNA synthesis

Chickpea organs (50 mg) were crushed to powder in liquid nitrogen using pestles and mortars and total RNA was isolated using RNAeasy Plant Mini kit (QIAGEN). Traces of DNA from RNA were removed by on-column DNAse treatment and the RNA was stored at -80°C. RNA concentration was estimated spectrophotometrically and RNA gel was also run from each batch of RNA to check the quality and verify the concentration. Reverse transcription was carried out (reaction volume 20 μl) using Omniscript RT kit (QIAGEN) as per the manufacturer’s instructions except for the quantity of RNA used. While the manufacturer’s recommended the use of 50 – 200 ng RNA per reverse transcription reaction (RT), the use of 50 ng RNA per RT yielded only about 20 bands per lane in the DDRT-PCR. The ideal number of bands per lane in DDRT-PCR should be about 50–60. Lowering the RNA concentration to 20 ng per RT yielded 50–60 bands per lane (data not shown). In all our experiments, 20 ng RNA per RT was used. The mRNA was reverse transcribed to first strand of cDNA using three independent reactions with three anchored poly T primers (AAGCTTTTTTTTTTTTTC, AAGCTTTTTTTTTTTTTG, AAGCTTTTTTTTTTTTTA).

### DDRT-PCR, electrophoresis and intensity analysis

Synthesis of the second strand and PCR was carried out in a volume of 25.0 μl using cDNA from 2 ng RNA [2 μl RT solution, 2.5 mM MgSO_4_, 0.1 mM of each dNTP’s mix, 0.8 μM of anchored and arbitrary primers (Sigma Aldrich, USA) and 1 U of Taq DNA polymerase (Life Tech)]. DDRT-PCR of treated and control anthers was carried out using 240 primer combinations (three anchored vs. 80 arbitrary primers, see Additional file 5 for list of primers) [45]. Amplifications were carried out in a Perkin Elmer Thermal Cycler (Gene Amp PCR System 9700) using 1 cycle of 4 min at 94°C followed by 39 cycles of 15 sec at 94°C (denaturation), 2 min at 40°C (primer annealing) and a 30 sec extension at 72°C followed by a cycle of 72°C for 8 min using the procedure as outlined by Liang *et al*. [46] with slight modifications. While Liang *et al*. [46] used fluorescence or radioactive labeling, we used silver staining.

The DDRT-PCR products were resolved on polyacrylamide gels (6%) and stained with silver nitrate as per Sambrook and Russell [47]. The gels were dried overnight at room temperature and scanned using hp scanjet 8200 scanner (HP) attached to a computer (Sony Vaio). The gel pictures were converted to TIFF files and differentially expressed bands were subjected to intensity analysis using Quantity one software (BioRad).

### Recovery, cloning and sequencing of differentially expressed cDNAs

For isolation of differentially expressed cDNAs, each band was eluted and re-amplified using the primers that were used to amplify the band in DDRT-PCR. The PCR conditions were the same as DDRT-PCR. Re-amplified products were separated on agarose gels (1.4%), extracted using QAIquick gel elution kit (QIAGEN) and cloned in pGEMT-Easy vector (Promega). The transformed vector was inserted into *Escherichia coli* strain DH5α. Ampicillin resistant clones were checked for insert and positive clones were sequenced.

### Sequence processing, gene annotation and functional categories

The EST sequences were checked for quality and analyzed by Seqman™ II 5.08 (DNASTAR, Inc. Lasergene Gene Corporation, Ann Arbor, MI) and VecScreen ( http://www.ncbi.nlm.nih.gov/VecScreen/VecScreen.html) to detect and remove pGEMT-Easy vector sequences. Manual sequence processing was also performed to confirm results. EST sequences, which were less than 75 bp long were removed. Duplicate entries were identified using DNASTAR, NCBI-BLAST ( http://blast.ncbi.nlm.nih.gov/Blast.cgi) and manually. ESTs were assembled into contigs using default parameters of CAP3 [48]. Gene annotation for identification and putative function was performed using NCBI-BLAST ( http://blast.ncbi.nlm.nih.gov/Blast.cgi). The CC, BP and MF of genes was determined by performing functional classification according to gene ontology ( http://www.geneontology.org/), UniProt Knowledge base ( http://www.uniprot.org/) and KEGG: Kyoto Encyclopedia of Genes and Genomes ( http://www.kegg.jp) after filtering the genes for more than two fold change and ≤0.05 P-value.

### RT-qPCR confirmation of candidate genes related to cold tolerance in anthers

Twenty five genes with different functions were selected to confirm their expression levels in anthers and spatio-temporal expression in anthers, gynoecium, leaves and roots using RT-qPCR. For RT-qPCR, gene-specific primers (Additional file 2) were designed from cDNA sequences using primer3Plus software ( http://www.bioinformatics.nl/cgi-bin/primer3plus/primer3plus.cgi). RNA for different experiments was isolated from untreated and cold treated organs at different time points as mentioned in the preceding section. First cDNA strand synthesis was carried out as outlined above. The cDNA synthesized using three anchored poly A primers (Additional file 5) was pooled in equal amounts and one μl of first strand cDNA mixture (1 ng RNA) was used for 12.5 μl RT-qPCR mixture. PCR was conducted in a thermal cycler (BioRad) at the following conditions: 5 min at 94°C followed by 30 cycles at 30 sec at 94°C, 30 sec at 52°C and 60 sec at 72°C and a final extension step of 72°C for 2 min. For normalization of RT-qPCR, *Actin ß* gene from chickpea (ACT1, EMBL-ACD37723.1) was used as reference. The PCR products were resolved in 1.4% agarose gel in tris acetate buffer at 120 v for 1 h and were visualized using GelRed™ Nucleic acid gel stain (Biotium, USA, 1 μl of 3x was added directly to the 12.5 μl PCR amplified mix) or ethidium bromide (Amresco, added to the gel @ 1 μl per 100 ml gel) in a UV transilluminator (Biorad).

### Data normalization and statistical analysis

After RT, initial concentration of the total cDNA in all the samples used for DDRT-PCR and RT-qPCR was normalized using the chickpea *Actin ß* gene. The CT value used was 30. The expression data (DDRT-PCR as well as RT-qPCR) were normalized to that of reference gene and normalized values were used to calculate fold change. All experiments were performed in two biological replicates and three technical replicates. Data were analyzed and graphs drafted using Microsoft Excel 3 (Microsoft, Redmond, USA). The means were expressed as arithmetic mean ± S.D.

### Accession numbers

The sequences of the transcripts are available under the accession numbers GenBank: JK998687 to JK998825.

## Electronic supplementary material

Additional file 1: **Identification and functional annotation of cold stress responsive anther transcripts.** (DOC 195 KB)

Additional file 2: **List of transcripts assessed by RT-qPCR and primer sequences.** (DOC 78 KB)

Additional file 3: **Regulation of cold stress responsive genes in anthers of ICC16349 at different time points.** (PPT 389 KB)

Additional file 4: **Spatial and temporal expression of cold stress responsive transcripts in anthers, gynoecium, leaves and roots of a tolerant chickpea line ICC16349.** (PPT 232 KB)

Additional file 5: **The sequences of forward and reverse primers used for DDRT PCR.** (DOC 69 KB)
