# Supplementary material for: Cold stress alters transcription in meiotic anthers of cold tolerant chickpea (Cicer arietinum L.)
Source: BMC Res Notes. 2014 Oct 11;7:717. doi: 10.1186/1756-0500-7-717 (PMC4201710; doi:10.1186/1756-0500-7-717)
Supplement: Supplementary file 1 — Additional file 1: Identification and functional annotation of cold stress responsive anther transcripts. (DOC 195 KB) [file 13104_2013_3240_MOESM1_ESM.doc]

**Identification and functional annotation of cold stress responsive anther transcripts.** The genotype was IC16349. The transcripts were identified using DDRT-PCR. The identity of the genes was established using NCBI-BLASTN, and cellular components, molecular and biological function using gene ontology (<http://www.geneontology.org/>), NCBI-BLASTX, UniProt Knowledge base (<http://www.uniprot.org/>) and [KEGG: Kyoto Encyclopedia of Genes and Genomes](http://www.google.co.in/url?sa=t&rct=j&q=kegg&source=web&cd=1&sqi=2&ved=0CC8QFjAA&url=http%3A%2F%2Fwww.genome.jp%2Fkegg%2F&ei=kQcET9LXBueSiQfsyfWeAQ&usg=AFQjCNFgEkzlz9pM3rm6Q460hJLg9IDXcg) ([http://www.kegg.jp](http://www.kegg.jp/)) pathways.

**Up-regulated**

| **Transcript no.** | **Best hit (BLASTN) to EST database** | | **Best hit (BLASTX) to nr protein database** | | **Function** |
| --- | --- | --- | --- | --- | --- |
|  | **Homology** | **Accession no.** | **Homology** | **Accession no.** |  |
| A2 | Drought-stress, salinity stress, *Cicer arietinum* | [HO066279.1](http://www.ncbi.nlm.nih.gov/nucleotide/313089422?report=genbank&log$=nucltop&blast_rank=2&RID=1U0T9WD7016)  GR408247.1 | - | - | Unknown |
| A3 | - | - | Cation/H^+^ antiporter 14 (syn. AT1G06970), *Arabidopsis thaliana* | NP_172178.1 | BF: Ion transport  MF: Antiporter activity  CC: Membrane |
| A6 | - | - | - | - | Unknown |
| A7 | - | - | - | - | Unknown |
| A8 | - | - | - | - | Unknown |
| A10 | - | - | Peroxisomal ABC transporter,  *Medicago truncatula* | XP_003601968.1 | BF: Transport (fatty acids), Pollen tube elongation, ovule fertilization, and seeds germination  MF: Fatty acid oxidation  CC: Glyoxisomal membrane |
| A12 | - | - | Conserved hypothetical protein, *Ricinus communis* | XP_002514025.1 | Unknown |
| A14 | - | - | - | - | Unknown |
| A15 | Salinity stress, *C. arietinum* | GR406275.1 | - | - | Unknown |
| A18 | - | - | - | - | Unknown |
| A19 | - | - | - | - | Unknown |
| A20 | - | - | Cation efflux system protien, *Agrobacterium radiobactor* K84 | YP_002543585.1 | BF: Ion transport, transmembrane transport  MF: Copper ion binding  CC: Membrane |
| A22 | Drought stress, *C. arietinum* | [HO067807.1](http://www.ncbi.nlm.nih.gov/nucleotide/313080621?report=genbank&log$=nucltop&blast_rank=1&RID=24M9SX10013) | 40S ribosomal protein SA*, M. truncatula* | [XP_003638087.1](http://www.ncbi.nlm.nih.gov/protein/358348100?report=genbank&log$=prottop&blast_rank=1&RID=1MSGER0001N) | BF: Translation  MF: Ribonucleoprotein  Cellular component: Cytoplasm |
| A24 | - | - | - | - | Unknown |
| A28 | - | - | - | - | Unknown |
| A34-1 | - | - | - | - | Unknown |
| A36-2 | Cdna,  *Lotus japonicas* | GO020417.1 | Putative beta-galactosidase, *M. truncatula* | XP_003607189.1 | BF: Metabolism: carbohydrate MF: beta-galactosidase activity  CC: Apoplast |
| A38-1 | Salinity stress, *C. arietinum* | [GR408029.1](http://www.ncbi.nlm.nih.gov/nucleotide/241808845?report=genbank&log$=nucltop&blast_rank=1&RID=24KPW88G016) | - | - | Unknown |
| A39-1 | - | - | - | - | Unknown |
| A58 | Drought-stress, salinity-stress, *C. arietinum* | FE671909.1  [GR404601.1](http://www.ncbi.nlm.nih.gov/nucleotide/241799300?report=genbank&log$=nucltop&blast_rank=3&RID=242UHX1101S) | - | - | Unknown |
| A59-1 | - | - | - | - | Unknown |
| A59-2 | Drought stress, *C. arietinum* | FL512459.1 | Glycerol kinase, *Glycine max* | NP_001237303.1 | BF: Metabolism (Glycerolipid metabolism)  MF: Glycerol kinase activity  CC: Unknown |
| A60 | - | - | Pectin methylesterase, *M. truncatula* | XP_003595372.1 | BF: Metabolism (cell wall modification, pollen tube growth)  MF: Pectin methylestrase activity  CC: Cell wall |
| A61-1 | Trichome isolated from the stem, *Medicago sativa* | EX522818.1 | ATP synthase subunit alpha, *M. truncatula* | XP_003588326.1 | Unknown` |
| A66 | Drought-stress, salinity-stress, *C. arietinum* | [GR406927.1](http://www.ncbi.nlm.nih.gov/nucleotide/241807184?report=genbank&log$=nucltop&blast_rank=43&RID=2416BKWY01N)  [FE671687.1](http://www.ncbi.nlm.nih.gov/nucleotide/169746567?report=genbank&log$=nucltop&blast_rank=92&RID=2416BKWY01N) | Protein MTR_3g035620, *M. truncatula,* | XP_003599574.1 | Unknown |
| A67 | - | - | Cysteine-rich receptor-like protein kinase, *M. truncatula* | XP_003589476.1 | BF: Signal transduction  MF: Protein serine/threonine kinase activity  CC: Unknown |
| A68 | - | - | - | - | Unknown |
| A69-3 | cDNA, *Solanum melongena* | [FS052767.1](http://www.ncbi.nlm.nih.gov/nucleotide/261702864?report=genbank&log$=nucltop&blast_rank=1&RID=UJ0XGJGZ013) | Mitochondrial protein, putative, *M. truncatula* | [XP_003588355.1](http://www.ncbi.nlm.nih.gov/protein/357436159?report=genbank&log$=prottop&blast_rank=1&RID=UJ0MA2SE01N) | Unknown |
| A71-2 | - | - | - | - | Unknown |
| A76 | Salinity-stress, biotic stress, *C. arietinum* | GR405290.1  [GR473693.1](http://www.ncbi.nlm.nih.gov/nucleotide/251744846?report=genbank&log$=nucltop&blast_rank=1&RID=2405PMDX01S) | - | - | Unknown |
| A78 | Drought-stress, *C. arietinum* | FE67109.1 | - | - | Unknown |
| A79 | - | - | - | - | Unknown |
| A80 | Drought-stress, *C. arietinum* | [FE671166.1](http://www.ncbi.nlm.nih.gov/nucleotide/169747772?report=genbank&log$=nucltop&blast_rank=2&RID=VM9BRJF7016) | - | - | Unknown |
| A81 | Developing flower, *Medicago truncatula* | BQ149654.1 | Protein kinase Serine/threonine, *A. thailana* | CAA16700.1 | BF: Signal transduction  MF: Protein serine/threonine kinase activity  CC: Nucleus |
| A82-1 | - | - | Heavy metal efflux pump CzcA, *Gamma proteobacterium* | ZP_05061697.1 | BF: unknown  MF: Transporter activity  CC: Unknown |
| A84-1 | Drought-stress, *C. arietinum* | FE673103.1 | Unknown, *M. truncatula* | ACJ85965.1 | Unknown |
| A97-1 | - | - | - | - | Unknown |
| A97-2 | - | - | Ralf-like 19 protein, ,  *A. thailana* | NP_850219.1 | BF: Signal transduction  MF: Unknown  CC: Unknown |
| A98-2 | Drought-stress, salinity-stress, biotic stress, *C. arietinum* | GR406292.1,  [FE672693.1](http://www.ncbi.nlm.nih.gov/nucleotide/169747511?report=genbank&log$=nucltop&blast_rank=2&RID=228EVD3V016),  [ES560228.1](http://www.ncbi.nlm.nih.gov/nucleotide/207112183?report=genbank&log$=nucltop&blast_rank=3&RID=228EVD3V016) | Hydrolase, *Zea mays* | NP_001150070.1 | BF: Unknown  MF: Hydrolase activity  CC: Unknown |
| A99-1 | - | - | Microspore-specific promoter2, *A. thaliana* | NP_568669.1 | BF: Pollen development  MF: Transcription  CC: Chloroplast |
| A101 | - | - | Pectinesterase, *G. max* | [XP_003591164.1](http://www.ncbi.nlm.nih.gov/protein/357441773?report=genbank&log$=prottop&blast_rank=8&RID=1MYW8HFP01N) | BF: Metabolism (cell wall modification, pollen tube growth)  MF: Pectin methylestrase activity  CC: Cell wall |
| A102-2 | cDNA, *M. truncatula* | [CB892520.1](http://www.ncbi.nlm.nih.gov/nucleotide/30099688?report=genbank&log$=nucltop&blast_rank=1&RID=227YDSM101S) | Aconitate hydratase,  *M. truncatula* | XP_003612247.1 | BF: Carbohydrate Metabolism,  Response to stress  MF: Iron sulfer cluster binding  CC: Cytoplasm |
| A103-1 | cDNA, *Linum usitatissimum* | [JG063627.1](http://www.ncbi.nlm.nih.gov/nucleotide/324896732?report=genbank&log$=nucltop&blast_rank=1&RID=227S6JNX014) | Conserved hypothetical protein, *R. communis* | XP_002516625.1 | Unknown |
| A104-1 | - | - | - | - | Unknown |
| A104-2 | - | - | SYP124 (SYNTAXIN OF PLANTS); SNAP receptor,  *M. truncatula* | XP_003593444.1 | BF: Vasicular mediate transport, Intracellular Protein transport, pollen development  MF: SNAP receptor activity  CC: Membrane |
| A105 | - | - | - | - | Unknown |
| A106 | - | - | - | - | Unknown |
| A107 | - | - | - | - | Unknown |
| A107-2 | - | - | - | - | Unknown |
| A108-2 | Drought-stress,  *C. arietinum* | [HO065695.1](http://www.ncbi.nlm.nih.gov/nucleotide/313086918?report=genbank&log$=nucltop&blast_rank=1&RID=22725W1E014) | - | - | Unknown |
| A111-1 | Drought-stress, *C. arietinum* | GR394671.1 | - | - | Unknown |
| A116-3 | Shoot apical meristem, *Pisum sativum* | FG536869.1 | L-ascorbate oxidase like protein, *M. truncatula* | XP_003611827.1 | BF: Ion transport  MF: Copper ion binding  CC: Unknown |
| A119 | - | - | - | - | Unknown |
| A120-1 | - | - | - | - | Unknown |
| A120-2 | - | - | Serine/threonine protein kinase, *M. truncatula* | XP_003618563.1 | BF: Signal transduction  MF: Protein Serine/Threonine Kinase Activity  CC: Unknown |
| A121-1 | - | - | AT5G57110 (Ca^2+^ transporting ATPase),  *A. thailana* | BAH20100.1 | BF: Calcium transport, ATP biosynthetic process  MF: Ca ion trnasmembrane transport,  Calmodulin binding  CC: Membrane |
| A123-1 | Leaf Cdna, *Lathyrus odoratus* | GO315513.1 | Potassium channel tetramerization domain-containing protein,  *R. communis* | XP_002509821.1 | BF: Unknown  MF: Voltage-gated potassium channel activity  CC: Membrane |
| A125-1 | cDNA, *G. max* | BU547359.1 | F16A14.19,  *A. thailana* | AAF79412.1 | BF: Transport  MF: Anion channel activity  CC: Unknown |
| A126-1 | Drought-stress, salinity-stress, biotic stress*, C. arietinum* | GR399076.1  GR407596.1  [DY475111.1](http://www.ncbi.nlm.nih.gov/nucleotide/105635944?report=genbank&log$=nucltop&blast_rank=5&RID=2258C3PD014) | Wound responsive protein,  *Phaseolus vulgaris* | [Q09020.1](http://www.ncbi.nlm.nih.gov/protein/1172597?report=genbank&log$=prottop&blast_rank=5&RID=1N2V4UX501N) | BF: Defense  MF: Unknown  CC: Cytosol |
| A132-1 | - | - | F-box family-6, *M. truncatula* | XP_003608851.1 | Unknown |
| A140-2 | - | - | Cyclin-dependent kinase CDC2C,  *M. truncatula* | XP_003621316.1 | BF: Signal transduction  Pollen tube growth  MF: Serine/threonine protein kinase activity  CC: Unknown |
| A141-1 | - | - | Plant-specific domain TIGR01570 family protein*, M. trunctatula* | XP_003602481.1 | Unknown |
| A142-1 | - | - | - | - | Unknown |
| A142-2 | - | - | - | - | Unknown |
| A146-1 | - | - | - | - | Unknown |
| A147-2 | - | - | - | - | Unknown |
| A154-1 | cDNA,  *M. truncatula* | CA920567.1 | - | - | Unknown |
| A157-5 | - | - | - | - | Unknown |
| A159-1 | Drought-stress, salinity-stress, *C. arietinum* | GR408576.1  FE671609.1 | - | - | Unknown |
| A161-1 | Salinity-stress, *C. arietinum* | GR406275.1 | - | - | Unknown |
| A162-2 | - | - | - | - | Unknown |
| A165-1 | - | - | - | - | Unknown |
| A166-1 | Drought-stress, *C. arietinum* | [HO066831.1](http://www.ncbi.nlm.nih.gov/nucleotide/313089998?report=genbank&log$=nucltop&blast_rank=1&RID=222HG6GR01S) | - | - | Unknown |
| A168-2 | Drought-stress, *C. arietinum* | GR394681.1 | - | - | Unknown |
| A171-2 | - | - | - | - | Unknown |
| A172-1-1 | - | - | - | - | Unknown |
| A178-1 | - | - | - | - | Unknown |
| AC39GA2 | cDNA, *Lotus japonicus* | FS350377.1 | Translation Intiation factor EIF-2B epsilon,  *M. truncatula* | XP_003618849.1 | BF: Translation  MF: Translation initiation factor activity  CC: Cytosol |
| AC39GA3 | - | - | - | - | Unknown |
| AC40GB2 | cDNA, *C. arietinum* | [FE670760.1](http://www.ncbi.nlm.nih.gov/nucleotide/169745544?report=genbank&log$=nucltop&blast_rank=1&RID=221GCY84016) | - | - | Unknown |
| AC42GE4 | - | - | - | - | Unknown |
| AC44GA 2 | Salinity-stress, *C. arietinum* | GR404294.1 | Putative sucrose phosphorylase, *Vibrio harveyi* HY01 | ZP 01985256.1 | BF: Carbohydrate metabolism (starch and sucrose metabolism)  MF: Cation binding, Sucrose phosphorylase activity  CC: Unknown |
| AC47G E1 | Drought stress, biotic stress, *C. arietinum* | [HS108089.1](http://www.ncbi.nlm.nih.gov/nucleotide/366460604?report=genbank&log$=nucltop&blast_rank=1&RID=21UH630M01S), GR 912650.1 | SRCI, *Glycine max* | BAA19768.1 | BF: Cold stress regulation  MF: Transcription  CC: Unknown |
| AC48GH2 | cDNA, *Phaseolus vulgaris* | [GW888949.1](http://www.ncbi.nlm.nih.gov/nucleotide/312062462?report=genbank&log$=nucltop&blast_rank=1&RID=21U74MN4014) | - | - | Unknown |
| AC48GI2 | cDNA, *Phaseolus acutifolius* | HO783882.1 | Chloroplast RF21, *Cicer arietinum* | YP_002149774.1 | BF: Unknown  MF: Unknown  CC: Chloroplast |
| AC50GC1 | Drought stress, *C. arietinum* | GR397920.1 | - | - | Unknown |
| AC50CG4 | Leave cDNA library,  *C. arietinum* | FE669846.1 | - | - | Unknown |
| AC51GA2 | Biotic stress, *C. arietinum* | [DY475167.1](http://www.ncbi.nlm.nih.gov/nucleotide/105636109?report=genbank&log$=nucltop&blast_rank=1&RID=TFXF3NEH011) | - | [-](http://www.ncbi.nlm.nih.gov/protein/294655785?report=genbank&log$=prottop&blast_rank=1&RID=TFXEYGN4014) | Unknown |
| AC52GD1 | cDNA, Trifolium pratense | BB906047.1 | Protein WAX2, *M. truncatula* | XP_003606194.1 | BF: Pollen sperm cell differentiation  MF: Iron ion binding,  Fatty acid biosynthetic process  CC: Integral to membrane |
| AN59CA2 | EST, *Pisum sativum* | [FG529507.1](http://www.ncbi.nlm.nih.gov/nucleotide/261231899?report=genbank&log$=nucltop&blast_rank=1&RID=TFSNK8DD015) | Casein kinase, *Ricinus communis* | XP_002516524.1 | BF: Signaling transduction  MF: ATP binding  CC: Unknown |

**Down-regulated**

| **Gene name** | **Best hit (BLASTN) to EST database** | | **Best hit (BLASTX) to nr protein database** | | **Function** |
| --- | --- | --- | --- | --- | --- |
|  | **Homology** | **Accession no.** | **Homology** | **Accession no.** |  |
| A1 | - | - | - | - | Unknown |
| A5 | Salinity-stress, *C. arietinum* | GR406908.1 | - | - | Unknown |
| A21 | - | - | - | - | Unknown |
| A26 | Biotic stress,  *C. arietinum* | [GR915690.1](http://www.ncbi.nlm.nih.gov/nucleotide/257706021?report=genbank&log$=nucltop&blast_rank=1&RID=24M4R3MD016) | - | - | Unknown |
| A27-1 | - | - | - | - | Unknown |
| A27-2 | - | - | - | - | Unknown |
| A40-2 | - | - | - | - | Unknown |
| A57 | - | - | Pescadillo-like protein, *Medicago truncatula* | XP_003625519.1 | Unknown |
| A62 | Drought-stress, *C. arietinum* | GR397782.1 | - | - | Unknown |
| A64-1 | - | - | Early nodulin-like protein, *M. truncatula* | XP_003609073.1 | BF: Pollen development  MF: Copper ion binding  CC: Membrane |
| A65 | - | - | protein MTR_3g086220, *M. truncatula* | XP_003601863.1 | Unknown |
| A70 | - | - | - | - | Unknown |
| A71-1 | - | - | Cell division cycle and apoptosis regulator protein, *M. truncatula* | XP_003613873.1 | BF: Cell division  MF; Unknown  CC: Unknown |
| A72 | Salinity-stress, *C. arietinum* | GR402153.1 | - | - | Unknown |
| A73 | Salinity-stress, drought stress, immune response to wilt, *C. arietinum* | GR407119.1  FL512452.1  GR913763.1 | 60S ribosomal protein L27a-3, *M. truncatula* | XP_003613127.1 | BF: Flower development  MF: Translation, Structural constituent of Ribosome  CC: Ribosome |
| A77-2 | Drought-stress, Salinity-stress,  *C. arietinum* | GR396634.1  GR408202.1 | Hypothetical protein NitaMp027, *Nicotiana tabacum* | YP_173374.1 | Unknown |
| A85-2 | Drought-stress, *C. arietinum* | FE673164.1 | - | - | Unknown |
| A87-1 | Biotic-stress,  *C. arietinum* | [GW421175.1](http://www.ncbi.nlm.nih.gov/nucleotide/315688553?report=genbank&log$=nucltop&blast_rank=1&RID=23ZDAAY601S) | - | - | Unknown |
| A89-2 | Drought-stress, *C. arietinum* | [FE671505.1](http://www.ncbi.nlm.nih.gov/nucleotide/169748051?report=genbank&log$=nucltop&blast_rank=2&RID=23Z9N9VC01N) | - | - | Unknown |
| A93-1 | cDNA, *Trifolium pretense* | BB919978.1 | - | - | Unknown |
| A93-2 | - | - | - | - | Unknown |
| A95-1 | Drought-stress, *C. arietinum* | FE672889.1 | - | - | Unknown |
| A109 | Drought-stress, Salinity-stress,  *C. arietinum* | GR407932.1  GR396593.1 | Mitochondrial protein, *M. truncatula* | XP_003588355.1 | Unknown |
| A114 | - | - | RPP1, *M. truncatula* | AB1511616.1 | BF: Defense, Resistance to peronospora parasitica  MF: Unknown  CC: Unknown |
| A115 | - | - | - | - | Unknown |
| A118 | - | - | ABC transporter family, *M. truncatula* | XP_003590459.1 | BF: Unknown  MF: Transporter activity  CC: Plasmodesmata |
| A130-2 | Cdna, *Lens culinaris* | [GT619264.1](http://www.ncbi.nlm.nih.gov/nucleotide/268539815?report=genbank&log$=nucltop&blast_rank=2&RID=224TZ570014) | - | - | Unknown |
| A134-1 | Drought-stress, *C. arietinum* | EH059119.1 | - | - | Unknown |
| A152-2 | cDNA, *M. truncatula* | CA919155.1 | - | - | Unknown |
| A155-1 | Biotic stress, drought stress, *C. arietinum* | DY475250.1  [HO066658.1](http://www.ncbi.nlm.nih.gov/nucleotide/313089801?report=genbank&log$=nucltop&blast_rank=2&RID=223J1ANJ016) | - | - | Unknown |
| A156-2 | - | - | - | - | Unknown |
| A168-1 | Drought-stress, *C. arietinum* | [GR394681.1](http://www.ncbi.nlm.nih.gov/nucleotide/241786969?report=genbank&log$=nucltop&blast_rank=1&RID=2229TT1N01N) | - | - | Unknown |
| A170-1 | - | - | Uncharacterized protein LOC100805236, Glycine ma] | XP_003523787.1 | Unknown |
| AC41GF1 | Drought-stress, *C. arietinum* | GR408975.1 | 60S ribosomal protein L34,  *M. truncatula* | XP_003621181.1 | BF: Translation  MF: Ribnucleoprotein  CC: Large subunit of ribosome |
| AC42GB3 | - | - | - | - | Unknown |
| AC45GA3 | Salinity-stress, Drought-stress, biotic-stress, *C. arietinum* | GR406281.1  [GR393869.1](http://www.ncbi.nlm.nih.gov/nucleotide/241792587?report=genbank&log$=nucltop&blast_rank=3&RID=21UP9RBY014)  [GR464273.1](http://www.ncbi.nlm.nih.gov/nucleotide/251739894?report=genbank&log$=nucltop&blast_rank=6&RID=21UP9RBY014) | ATPase subunit 8, *Lotus japonicus* | YP_005090498.1 | BF: Energy (ATP synthesis coupled proton transport, (Mitochondria)  MF: Hydrogen ion transmembrane transporter activity  CC: Mitochondria |
| AC53GA1 | - | - | - | - | Unknown |
