# Supplementary material for: Cold stress alters transcription in meiotic anthers of cold tolerant chickpea (Cicer arietinum L.)
Source: BMC Res Notes. 2014 Oct 11;7:717. doi: 10.1186/1756-0500-7-717 (PMC4201710; doi:10.1186/1756-0500-7-717)
Supplement: Supplementary file 2 — Additional file 2: List of transcripts assessed by RT-qPCR and primer sequences. (DOC 78 KB) [file 13104_2013_3240_MOESM2_ESM.doc]

**List of transcripts assessed by RT-qPCR and primer sequences.**

**List of transcripts**

| **Sr. no.** | **TC number** | **Homology** | **Sr. no.** | **TC number** | **Homology** |
| --- | --- | --- | --- | --- | --- |
| **Pollen development** |  |  | **Defense** |  |  |
| 1 | A10 | Peroxisomal ABC transporter,  *M. truncatula*, XP_003601968.1 | 11 | A114** | RRP1, *Medicago truncatula*, AB1511616.11 |
| 2 | A60 | Pectin methylesterase, *M. truncatula*, XP_003595372.1 | 12 | A126-1* | Wound responsive protein,  *Phaseolus vulgaris*, Q09020.1 |
| **Carbohydrate metabolism** |  |  | **Translation** |  |  |
| 3 | A36-2 | Beta-galactosidase, *Arabidopsis thaliana*, CAB64750.1 | 13 | A22 | 40S ribosomal protein SA, *M. truncatula*, XP_003638087.1 |
| 4 | A59-2 | Glycerol kinase, *Glycine max*, NP_001237303.1 | 14 | AC39GA2 | Translation Intiation factor EIF-2B epsilon, *M. truncatula,* XP_003618849.1 |
| 5 | AC44GA 2 | Sucrose phosphorylase, *Vibrio harveyi* HY01, ZP 01985256.1 | 15 | AC41GF1 | 60S ribosomal protein L34, *M. truncatula,* XP_003621181.1 |
| **Transport** |  |  | **Unknown** |  |  |
| 6 | A20 | Cation efflux system protien, *Agrobacterium radiobactor* K84, YP_002543585.1 | **16** | A58 | No homology |
| 7 | A118 | ABC transporter family, *M. truncatula*, XP_003590459.1 | 17 | A62 | No homology |
| **Signal transduction** |  |  | **18** | A71-2 | No homology |
| 8 | A140-2 | Cyclin-dependent kinase CDC2C,  *M. truncatula*, XP_003621316.1 | 19 | A77-2 | No homology |
| **Cell division** |  |  | 20 | A84-1 | No homology |
| 9 | A71-1 | Cell division cycle and apoptosis regulator protein, *M. truncatula*, XP_003613873.1 | 21 | A95-1 | No homology |
| **Transcription** |  |  | 22 | A108-2 | No homology |
| 10 | AC 47GE1 | SRCI, *Glycine max*, BAA19768.1 | 23 | A155-1 | No homology |
|  |  |  | 24 | A166-1 | No homology |
|  |  |  | 25 | A170-1 | No homology |

**Primer sequences**

| **TC no** | **Sequence (5′-3′)** | | **Gene** | **Sequence (5′-3′)** | |
| --- | --- | --- | --- | --- | --- |
|  | **Forward primers** | **Reverse primer** |  | **Forward primers** | **Reverse primer** |
| A10 | AGAATGAATGGTTGACAAGAGGA | CTTTTGATTCCGTGCTGCTT | A108-2 | TTGGAAGAGGTCAGTACAATATTTGG | TTCAAAATGAATCCCCGTGA |
| A20 | CACCTCCCACTCCAATCACT | CATACACCAAACAACCCATCC | A114 | CAGCAGCAAACATCAGAATGA | CAGTCGCAAGCAATTGAGTC |
| A22 | CCAAACATCTCAGCCCATAAA | AAGCAACTGGCTGGGAATAA | A118 | TCGTGTAGCTAGAACTTGATCAGAA | TTACAGCAGGATGGGTTCTACT |
| A36-2 | GCCAGACATGGAGGCTTAAA | TCCGTGATTCCATTCAGCTT | A126-1 | GATTCAAGGCCAACGAGAAC | TGTCAAAGCCAAACAACCAA |
| A58 | TGTTAAATCCCAAGCCCAAA | GGGTGTTGTATGAAATTGAGGTC | A140-2 | GGCAGGCTTGATGGATTACA | CAAGCTTCCTCATGCAACAA |
| A59-2 | TTGATGCAGATTCAGGCAGA | CCAGCGTCCTTCAGTTTTTC | A155-1 | CCTAAGCTTTTCGGTGATTCC | TTTAGATCTTTTTCCGCCATC |
| A60 | TTCAAGCAGTGAACTCAAGTTGT | AGTGGATTGGACGCAAAGAT | A166-1 | CCTAAGCTTTTCGGTGATTCC | TTTAGATCTTTTTCCGCCATC |
| A62 | TGGAAGTGCTATGGATTTGG | CCTATCTCTGTCACGCAAGC | A170-1 | CAGGGTCCTGAAATCCAATC | CGAGCAAATCATCAATTTCG |
| A71-1 | TGGAAGTGCTATGGATTTGG | CCTATCTCTGTCACGCAAGC | AC39GA2 | CCTCTTATGCCCCACAAAGA | AAGACGCAGATGAGTCGGATA |
| A71-2 | TGCCATTTAGAAAGGTGTGAGA | ACATGTCCAGGATATTGCATCT | AC41GF1 | TTTGGATCTTCAATACCTTCTTCAC | CTTGAAAGAGGTCTAGATTGTCTCG |
| A77-2 | TACTAACGGGGTCTCGGTTG | GCAAAGGTCATGGGGAGATA | AC44GA 2 | TGTCAGAAGTTGGGTGAAGC | TGAAACCAGATGACCTTGGA |
| A84-1 | CCAATACCATCGACCTTCCA | TTTTCACGTTGGGCTTGAAT | AC 47G E1 | TTTTGATATTTTCACCCTCTCCA | TTTGCAAAGCCAGAACAACA |
| A95-1 | AACTATCACCTGATTGTGGCTAA | TTTTTCAGATCTCTGATGAATCG |  |  |  |
