# Supplementary material for: Cold stress alters transcription in meiotic anthers of cold tolerant chickpea (Cicer arietinum L.)
Source: BMC Res Notes. 2014 Oct 11;7:717. doi: 10.1186/1756-0500-7-717 (PMC4201710; doi:10.1186/1756-0500-7-717)
Supplement: Supplementary file 3 — Additional file 3: Regulation of cold stress responsive genes in anthers of ICC16349 at different time points. (PPT 389 KB) [file 13104_2013_3240_MOESM3_ESM.ppt]

## Slide 1
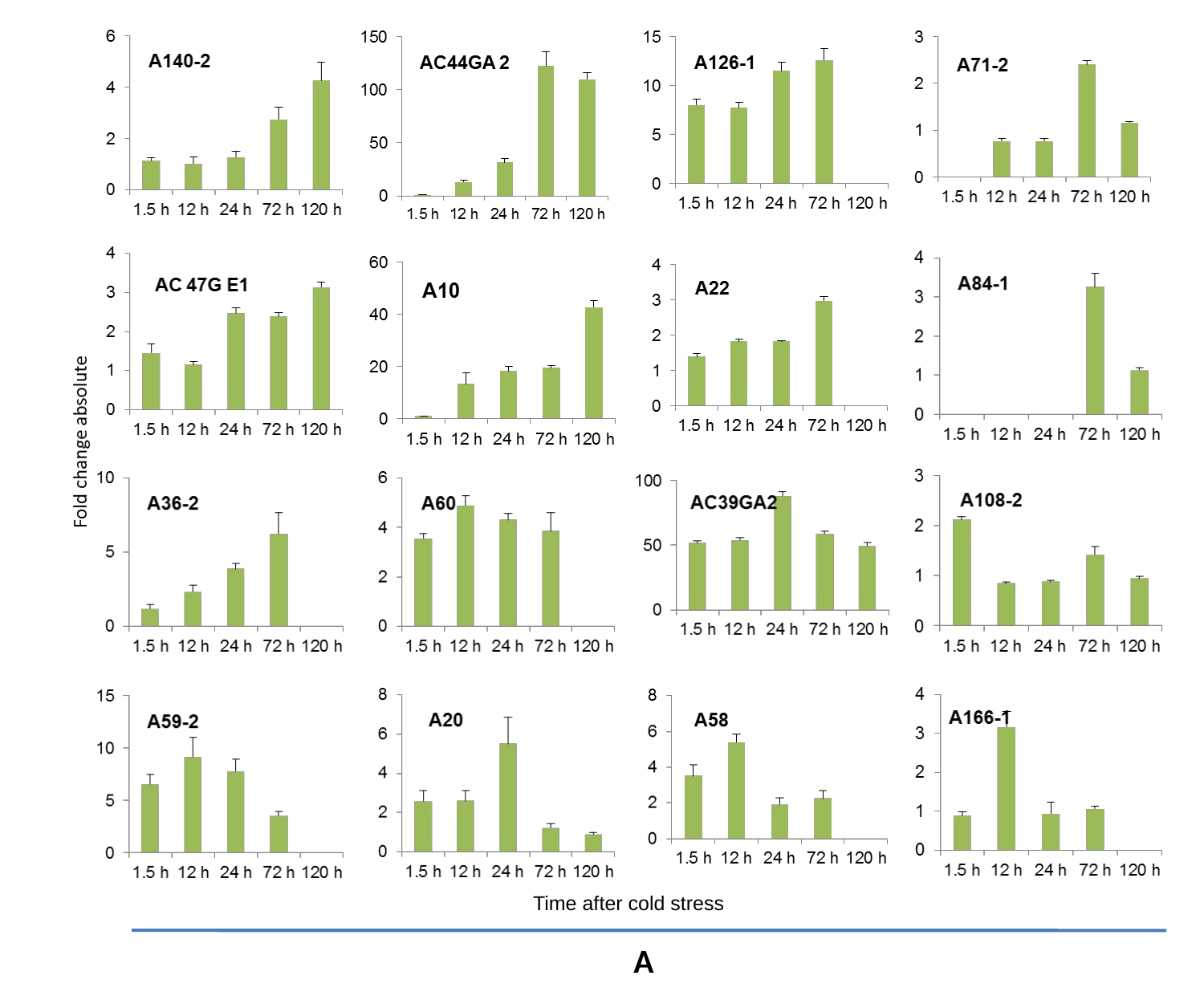

Time after cold stress
A

## Slide 2
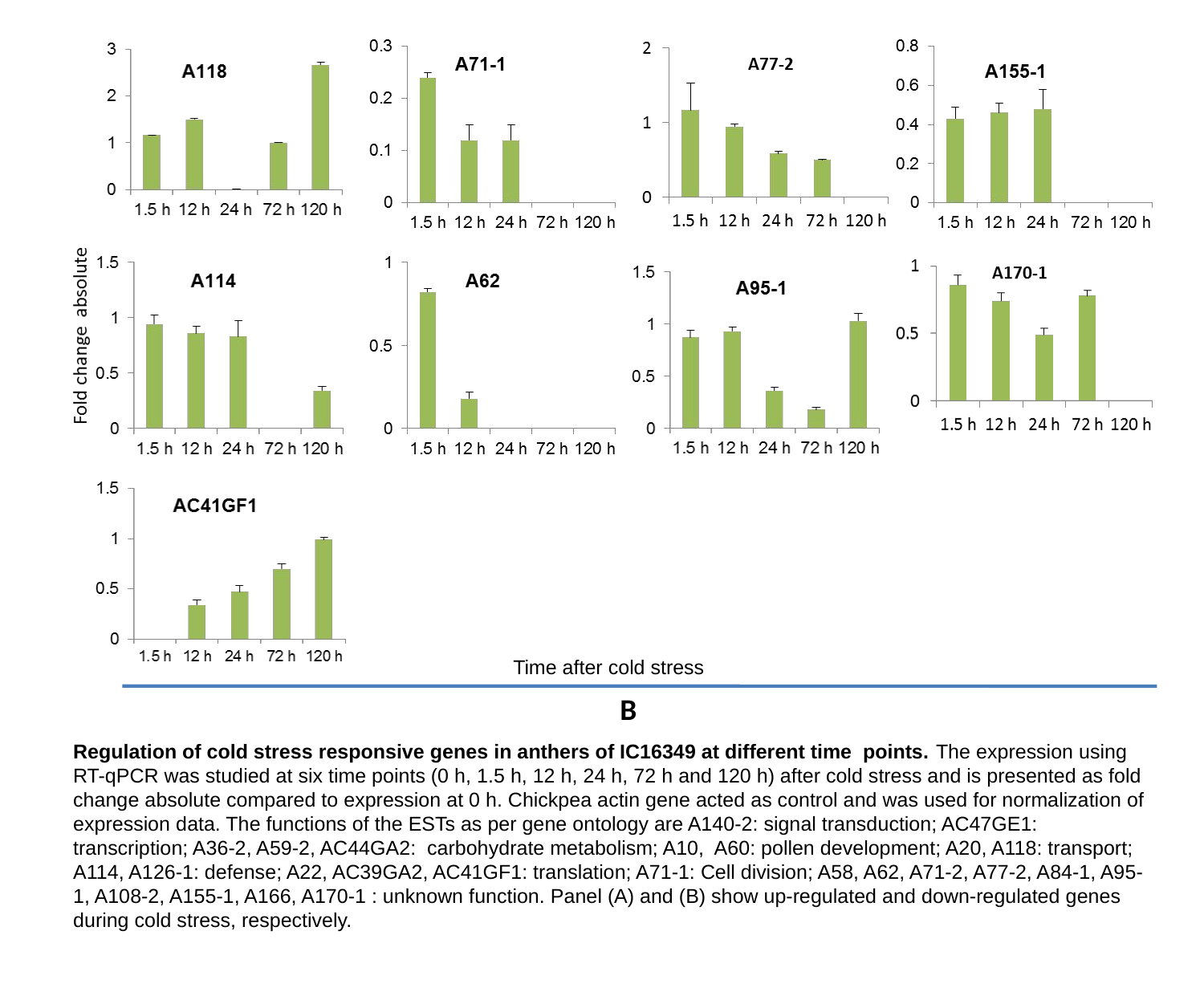

Time after cold stress
B
Regulation of cold stress responsive genes in anthers of IC16349 at different time points. The expression using RT-qPCR was studied at six time points (0 h, 1.5 h, 12 h, 24 h, 72 h and 120 h) after cold stress and is presented as fold change absolute compared to expression at 0 h. Chickpea actin gene acted as control and was used for normalization of expression data. The functions of the ESTs as per gene ontology are A140-2: signal transduction; AC47GE1: transcription; A36-2, A59-2, AC44GA2: carbohydrate metabolism; A10, A60: pollen development; A20, A118: transport; A114, A126-1: defense; A22, AC39GA2, AC41GF1: translation; A71-1: Cell division; A58, A62, A71-2, A77-2, A84-1, A95-1, A108-2, A155-1, A166, A170-1 : unknown function. Panel (A) and (B) show up-regulated and down-regulated genes during cold stress, respectively.

## Slide 3
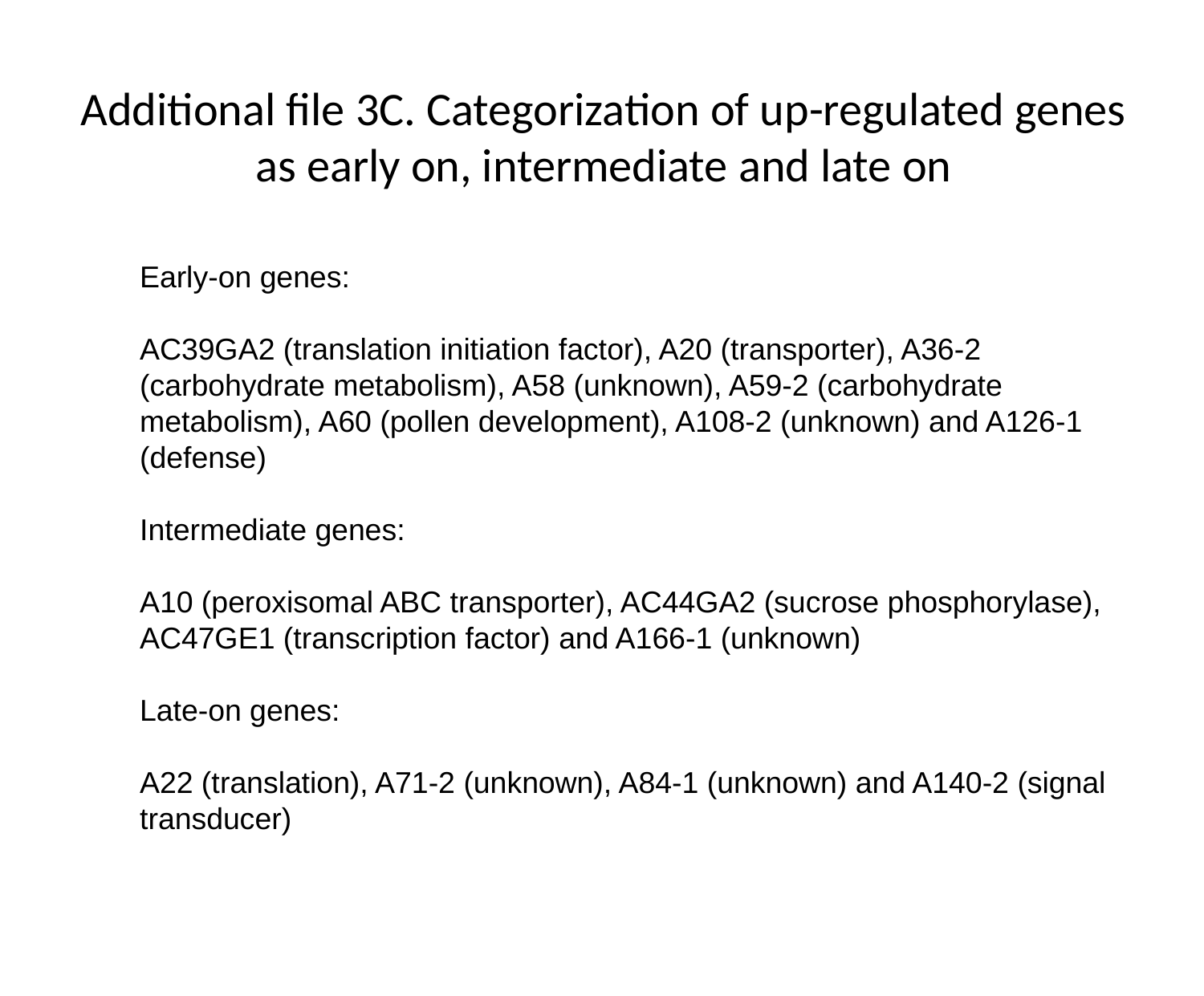

# Additional file 3C. Categorization of up-regulated genes as early on, intermediate and late on
Early-on genes:
AC39GA2 (translation initiation factor), A20 (transporter), A36-2 (carbohydrate metabolism), A58 (unknown), A59-2 (carbohydrate metabolism), A60 (pollen development), A108-2 (unknown) and A126-1 (defense)
Intermediate genes:
A10 (peroxisomal ABC transporter), AC44GA2 (sucrose phosphorylase), AC47GE1 (transcription factor) and A166-1 (unknown)
Late-on genes:
A22 (translation), A71-2 (unknown), A84-1 (unknown) and A140-2 (signal transducer)
