# Supplementary material for: Cold stress alters transcription in meiotic anthers of cold tolerant chickpea (Cicer arietinum L.)
Source: BMC Res Notes. 2014 Oct 11;7:717. doi: 10.1186/1756-0500-7-717 (PMC4201710; doi:10.1186/1756-0500-7-717)
Supplement: Supplementary file 4 — Additional file 4: Spatial and temporal expression of cold stress responsive transcripts in anthers, gynoecium, leaves and roots of a tolerant chickpea line ICC16349. (PPT 232 KB) [file 13104_2013_3240_MOESM4_ESM.ppt]

## Slide 1
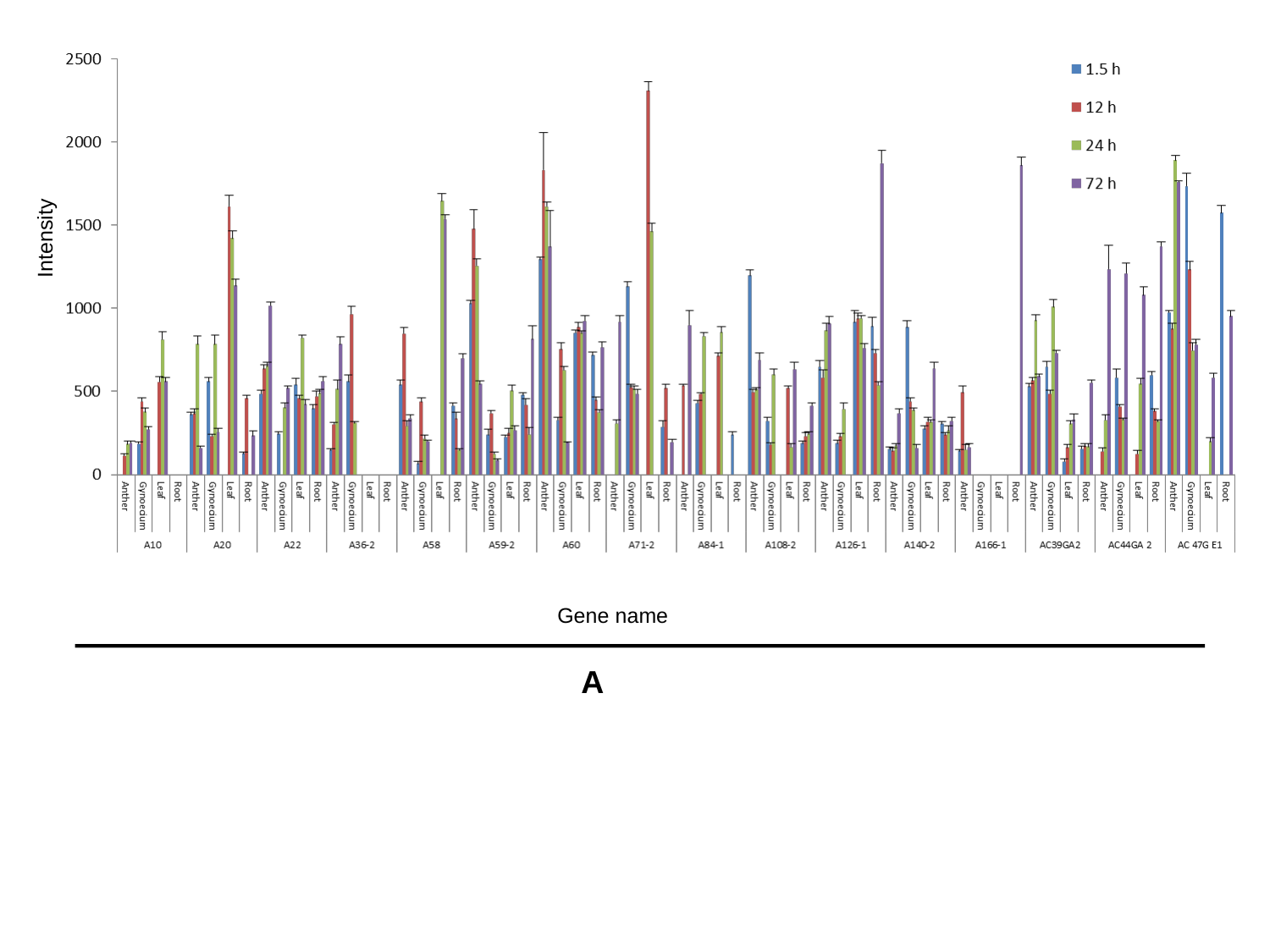

Intensity
Gene name
A

## Slide 2
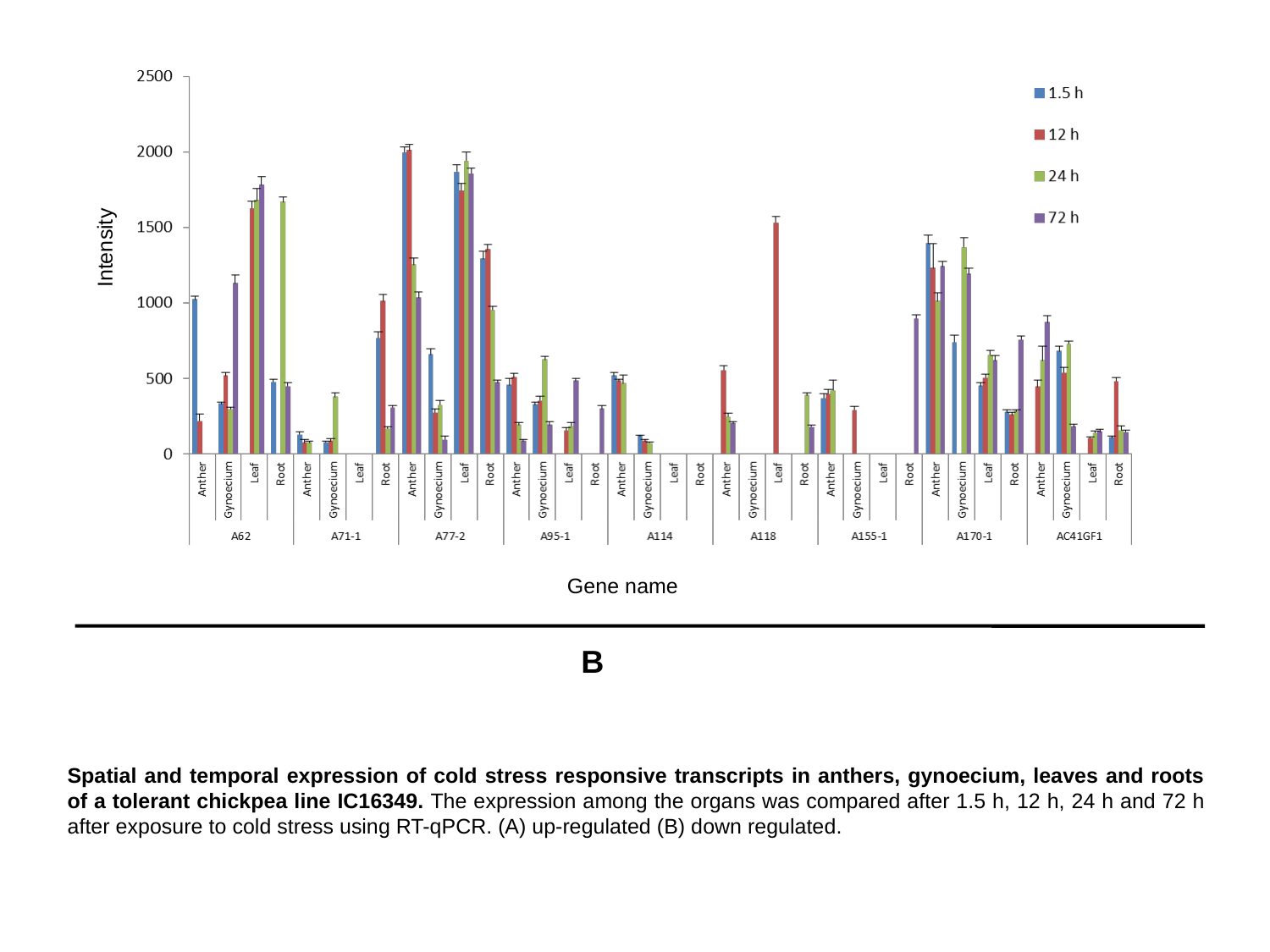

Intensity
Gene name
B
Spatial and temporal expression of cold stress responsive transcripts in anthers, gynoecium, leaves and roots of a tolerant chickpea line IC16349. The expression among the organs was compared after 1.5 h, 12 h, 24 h and 72 h after exposure to cold stress using RT-qPCR. (A) up-regulated (B) down regulated.
