# Supplementary material for: Cold stress alters transcription in meiotic anthers of cold tolerant chickpea (Cicer arietinum L.)
Source: BMC Res Notes. 2014 Oct 11;7:717. doi: 10.1186/1756-0500-7-717 (PMC4201710; doi:10.1186/1756-0500-7-717)
Supplement: Supplementary file 5 — Additional file 5: The sequences of forward and reverse primers used for DDRT PCR. (DOC 69 KB) [file 13104_2013_3240_MOESM5_ESM.doc]

**The sequences of forward and reverse primers used for DDRT PCR.** The sequence of forward primer is 5'-CTTNNXXXXXXXX-3' (N = A, C, G, or T). The 3' eight mRNA specific bases (XXXXXXXX) for the 5' primer are presented in the Table. The primers are as per Yang *et al*. (2006).

| **ID** | **Sequence (5'- 3')** | **ID** | **Sequence (5'- 3')** | **ID** | **Sequence (5'- 3')** |
| --- | --- | --- | --- | --- | --- |
| **Reverse primers** | |  |  |  |  |
| 1 | AAGCTTTTTTTTTTTTTC | 2 | AAGCTTTTTTTTTTTTTG | 3 | AAGCTTTTTTTTTTTTTA |
| **Forward primers** | |  |  |  |  |
| H-AP1 | TACTCCCT | H-AP28 | TCAAGGCT | H-AP55 | TTCAGCAC |
| H-AP2 | ATCTCCGA | H-AP29 | AGAAGGCT | H-AP56 | ATCAGCAG |
| H-AP3 | TCTTCCGA | H-AP30 | ATGAGGGA | H-AP57 | TACAGCAG |
| H-AP4 | TCATCCGA | H-AP31 | TCAAGGGA | H-AP58 | AGAAGCAG |
| H-AP5 | AGATCCGA | H-AP32 | AAGAGGGT | H-AP59 | AAGAGCTC |
| H-AP6 | GATTCCGT | H-AP33 | ATGAGGGT | H-AP60 | ATGAGCTC |
| H-AP7 | ATGTCCGT | H-AP34 | TTCAGGGT | H-AP61 | ATCAGCTC |
| H-AP8 | AACTCCGT | H-AP35 | TCTAGGGT | H-AP62 | TTGAGCTC |
| H-AP9 | GAATCCAC | H-AP36 | GATAGGAC | H-AP63 | ACAAGCTC |
| H-AP10 | TTCTCCAC | H-AP37 | CAAAGGAC | H-AP64 | TCAAGCTG |
| H-AP11 | ATCTCCAC | H-AP38 | GATAGGAG | H-AP65 | CATAGCTG |
| H-AP12 | GTTTCCAG | H-AP39 | CAAAGGAG | H-AP66 | CTTTGGCA |
| H-AP13 | ATCTCCAG | H-AP40 | TCTAGGAG | H-AP67 | AGATGGCA |
| H-AP14 | AAGTCCTC | H-AP41 | AAGAGGTC | H-AP68 | GAATGGCT |
| H-AP15 | GATTCCTC | H-AP42 | CAAAGGTC | H-AP69 | TCATGGCT |
| H-AP16 | ATGTCCTC | H-AP43 | AGAAGGTC | H-AP70 | AGATGGCT |
| H-AP17 | GTTTCCTC | H-AP44 | AAGAGGTG | H-AP71 | AGTTGGCT |
| H-AP18 | AACTCCTC | H-AP45 | ACAAGGTG | H-AP72 | CAATGGGT |
| H-AP19 | AGATCCTC | H-AP46 | AAGAGCCA | H-AP73 | TCTTGGGT |
| H-AP20 | TTCTCCTG | H-AP47 | CAAAGCCA | H-AP74 | ACATGGAC |
| H-AP21 | AACTCCTG | H-AP48 | ATCAGCCA | H-AP75 | CAATGGAG |
| H-AP22 | ATCAGGCA | H-AP49 | AGTAGCCA | H-AP76 | CTTTGGTC |
| H-AP23 | AAGAGGCT | H-AP50 | TTCAGCCT | H-AP77 | AGTTGGTC |
| H-AP24 | ATGAGGCT | H-AP51 | CTTAGCCT | H-AP78 | AAGTGGTG |
| H-AP25 | CAAAGGCT | H-AP52 | ACAAGCCT | H-AP79 | GAATGGTG |
| H-AP26 | TTGAGGCT | H-AP53 | AAGAGCGA | H-AP80 | AACTGGTG |
| H-AP27 | TCTAGGCT | H-AP54 | TACAGCGA |  |  |
